# A Review on Mechanical Performance of Hybrid Natural Fiber Polymer Composites for Structural Applications

**DOI:** 10.3390/polym13132170

**Published:** 2021-06-30

**Authors:** N. M. Nurazzi, M. R. M. Asyraf, S. Fatimah Athiyah, S. S. Shazleen, S. Ayu Rafiqah, M. M. Harussani, S. H. Kamarudin, M. R. Razman, M. Rahmah, E. S. Zainudin, R. A. Ilyas, H. A. Aisyah, M. N. F. Norrrahim, N. Abdullah, S. M. Sapuan, A. Khalina

**Affiliations:** 1 Institute of Tropical Forestry and Forest Products (INTROP), Universiti Putra Malaysia (UPM), Seri Kembangan 43400, Malaysia; mohd.nurazzi@gmail.com (N.M.N.); atiyah88@gmail.com (S.F.A.); shazra.shazleen@yahoo.com (S.S.S.); ayu.rafiqah@yahoo.com (S.A.R.); mmharussani17@gmail.com (M.M.H.); edisyam@upm.edu.my (E.S.Z.); 2 Centre for Defence Foundation Studies, Universiti Pertahanan Nasional Malaysia (UPNM), Kem Perdana, Sungai Besi, Kuala Lumpur 57000, Malaysia; 3 Department of Aerospace Engineering, Universiti Putra Malaysia (UPM), Seri Kembangan 43400, Malaysia; asyrafriz96@gmail.com; 4 Faculty of Applied Sciences, Universiti Teknologi MARA (UiTM), Shah Alam 40450, Malaysia; sitihasnahkam@uitm.edu.my (S.H.K.); rahmahmd@uitm.edu.my (M.R.); 5 Research Centre for Sustainability Science and Governance (SGK), Institute for Environment and Development (LESTARI), Universiti Kebangsaan Malaysia (UKM), Bangi 43600, Malaysia; mrizal@ukm.edu.my; 6 Department of Mechanical and Manufacturing Engineering, Faculty of Engineering, Universiti Putra Malaysia, Seri Kembangan 43400, Malaysia; 7 School of Chemical and Energy Engineering, Faculty of Engineering, Universiti Teknologi Malaysia (UTM), Johor Bahru 81310, Malaysia; 8 Research Centre for Chemical Defence, Universiti Pertahanan Nasional Malaysia (UPNM), Kem Perdana, Sungai Besi, Kuala Lumpur 57000, Malaysia

**Keywords:** hybrid composite, mechanical performance, natural fiber, polymer composite

## Abstract

In the field of hybrid natural fiber polymer composites, there has been a recent surge in research and innovation for structural applications. To expand the strengths and applications of this category of materials, significant effort was put into improving their mechanical properties. Hybridization is a designed technique for fiber-reinforced composite materials that involves combining two or more fibers of different groups within a single matrix to manipulate the desired properties. They may be made from a mix of natural and synthetic fibers, synthetic and synthetic fibers, or natural fiber and carbonaceous materials. Owing to their diverse properties, hybrid natural fiber composite materials are manufactured from a variety of materials, including rubber, elastomer, metal, ceramics, glasses, and plants, which come in composite, sandwich laminate, lattice, and segmented shapes. Hybrid composites have a wide range of uses, including in aerospace interiors, naval, civil building, industrial, and sporting goods. This study intends to provide a summary of the factors that contribute to natural fiber-reinforced polymer composites’ mechanical and structural failure as well as overview the details and developments that have been achieved with the composites.

## 1. Introduction

To date, global industries have considered the application of natural fibers as an alternative to synthetic materials as one of the constituents in the composites due to their advantages of being renewable and possessing marketing appeal in composites manufacturing industries. The unique properties of natural fibers—for instance, low cost, low density, recyclability, biodegradability, and sustainable in terms of resources as well as and abundant availability—make them considered as the materials of choice. In addition, in terms of natural fibers preparation, it is a facile process with little equipment needed, plus, the preparation approaches utilized do not generate harmful gases, which mitigates the environmental pollution. The utilization of the natural fibers derived from agricultural crops residue as the main source of the composites product can help reduce the open burning that can lead to air pollution; thus, it can help protect the environment [1,2,3]. The development of natural fibers-based composites has been considered to replace the applications of glass-fiber-based composites especially in many major industries related to structural applications including the civil construction industries, aerospace and automotive industries, etc. These composites-based products can be cost-effective materials for the production of automobile body panels, interiors, storage devices, building and industrial panels, etc. [4,5].

Despite all the advantages, the use of natural fibers as base materials in the composites system suffered from the disadvantages of the materials themselves. The fact that the water absorption of the natural fibers is higher than that of synthetic fibers has lowered its properties, thus imparting deterioration toward the composite’s properties as a whole [5]. Natural fibers inherited a hydrophilic character, which limits their success in polymer reinforcement. These hydrophilic properties resulted in high moisture absorption, poor matrix–fiber interfacial adhesion, and poor fiber dispersion. Natural fibers also showed high flammable properties, thus limiting their applications. However, these drawbacks can be overcome via surface treatment of the natural fibers. The treatment improves the surface adhesion of the natural fibers and enhances the bonding properties of fibers with the matrix by removing the impurities present on the surface of the fibers [2,3].

Over the past few years, there has been continuous development of improving the performance of the composites. The interest in hybridizing the materials in the composites in the common matrix has been growing since 2013. The hybrid composites were known to contain two or more fibers in one matrix. The fiber can be in the form of common particulate fibers, woven and non-woven fibers, and also nano-scale filler. This concept also applied to the blending of two or more polymers reinforced with one or more filler with similar types that has two or more sizes or dimensions. Hybridization creates the possibility of achieving a balance and more attractive characteristics and cost for the composite structure, which is difficult to obtain with a single kind of reinforcement. Research discovered that the behavior of hybrid composites seems to be simply a weighted sum of the individual components, which is controlled by factors such as the nature of the matrix; fiber length; chemical composition of the reinforcements; fiber–matrix interface; and hybrid design. In other words, by careful selection of reinforcements and processing techniques, it is possible for the researcher to meet various practical requirements with economic benefits [6,7,8].

Hybridization offers new opportunities to broaden the function of the composite materials, particularly in advanced applications by improving the toughness or impact resistance [2,9]. Hybrid composites also provide more design freedom compared to non-hybrid composites, which leads to a synergetic effect not possessed by any one material alone. The synergetic effect can be achieved via several aspects including selection of the fibers, suitable fiber combination, and their interaction in the hybrid system. For architects and engineers to design and handle the materials, the characteristics and properties of the materials need to be understood. The limitations of the materials need to be studied as well, as the hybridization might lead to deterioration of the composites performance [10]. Therefore, in order to understand the effects of hybridizing the natural fibers in the composites system, more information is needed through continuous studies of different effects between various types of natural fibers and their effects with the matrix polymer [11]. This information can be beneficial to be applied specially for the desired applications. Therefore, in this review, the information regarding the natural fibers and the effects of their utilization in the composites system were discussed. With this review, comprehensive information can be shared particularly on the effects of natural fibers-reinforced polymer composites for structural applications along with the recent trends of the materials. This enables the readers to realize several opportunities that have not been exploited yet, which can help with developing new and advanced technology in this field of research.

## 2. Natural Fiber

**Figure 1:**
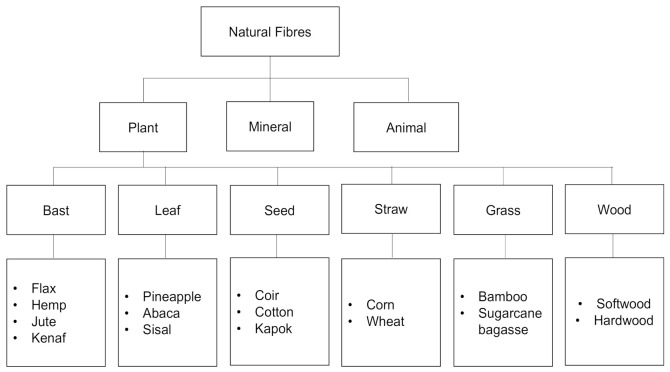
Schematic representations of natural fibers.

**Figure 2:**
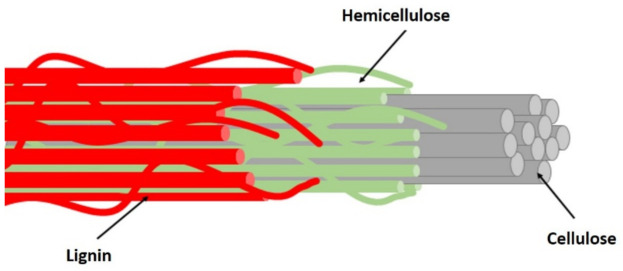
Basic structure of lignocellulosic fiber.

Natural fibers are non-synthetic, non-manmade fibers that may be derived from plants or animals. Natural fibers have long gained attention as reinforcing agents for polymer composites owing to their sustainability, ready availability, and satisfactory mechanical strength [12,13,14,15,16]. Natural fibers are generally classified into several categories based on their source, as shown in Figure 1. Among these fibers, plant-based fibers are the most often used in a variety of applications and have a high market value. The lignocellulosic component of plant biomass primarily consists of cellulose (C_6_H_10_O_5_)_n_, hemicellulose (C_5_H_8_O_4_)_m_, and lignin [C_9_H_10_O_3_(OCH_3_)_0.9−1.7_]_x_, which were incorporated to form a strong cellulose–hemicellulose–lignin complex within the plant material [17,18,19]. Figure 1 shows the interaction of cellulose, hemicellulose, and lignin in the fiber. Figure 2 shows the basic structure of lignocellulosic fiber consisted of lignin, hemicellulose and cellulose.

It is well known that chemical constituents of natural fibers significantly vary due to their diverse origins and types [20]. Growing and harvesting conditions can also influence this variability. Table 1 shows different lignocellulosic compositions, while Table 2 tabulated their physical and tensile properties in filament form for several natural fibers [12]. Generally, the cellulose, hemicellulose, and lignin in a typical lignocellulosic fall within the range of 30 to 60%, 20 to 40%, and 15 to 25%, respectively. These lignocellulosic compositions greatly influenced the mechanical properties of the fibers and resulted in significant properties for the mechanical performance of the polymer composites.

Cellulose is the most abundant component in biomass and finds applications in many spheres of modern industry [28,29,30,31,32,33]. Cellulose is a high molecular weight linear homopolymer, comprising β-d-glucopyranosyl repeating units joined by 1–4 glycosidic linkages. It can be found in a linear chain of anhydro-glucose monomer units connected by 1-4 β-linkages and stabilized at the end terminal with non-reducing and reducing sugar units. The properties of the cellulose chain can be attributed to reactive –OH groups that reside on position C-2, C-3, and C-6. It is noted that these hydroxyl groups and their ability to form hydrogen bonds play a major role in directing the crystalline packing and also govern the physical properties of cellulose. The hydrogen bonding of many cellulose molecules to each other results in the formation of microfiber that can interact to form fiber. Cellulose fibers are being used for several applications because of so many advantages such as being abundantly available, low weight, biodegradable, cheap, renewable, having a low abrasive nature, and exhibiting good mechanical properties.

Hemicellulose is considered as a second major component of lignocellulosic that consists of short chains of different polysaccharides such as xylan, galactomannan, glucuronoxylan, arabinoxylan, glucomannan, and xyloglucan that are held together by β-(1,4)- and/or β-(1,3)-glycosidic bonds. The degradation of hemicellulose into monosaccharides is due to its low degree of polymerization and non-crystalline nature; thereby, it is widely used in drug deliveries, hydrogels, and cosmetics applications. For lignin, it consists of a 3D cross-linked polymer that consists of phenyl propane structural units and varies depending on the substitution of the methoxyl groups present in the aromatic rings, which are linked to each other by aryl ether linkages, e.g., β-O-4, α-O-4 and carbon–carbon bonds, e.g., 5–5, β-β. Three basic units that constitute the lignin polymer are p-hydroxyphenyl (H), guaiacyl (G), and syringyl (S). Lignin functions as a protecting boundary by covalently linking to the cellulose and hemicellulose, which enhances the recalcitrance of the lignocellulosic biomass.

According to Ramamoorthy et al. [19], plant fibers can be classified into six types: bast, seed, leaf, straw, grass, and wood fibers. Bast fibers are made up of a bundle of tube-like cell walls and are obtained from the outer cell layers of the stems of various plants. This fiber has been applied in various industries including geotextiles and insulation. Moreover, the demand for polymer composites made from bast fibers has increased considerably in recent decades due to their outstanding properties such as reliable, low cost, lightweight, non-toxic, and structurally sound. Leaf fiber, also known as hard fiber, is obtained by scraping away non-fibrous material, and the resultant fiber is stiffer and coarser than bast fibers; hence, it has little market appeal [34]. The properties of the fibers may change over the course of the fiber depending on their length. Leaf fiber has a high cellulose content of up to 70%, but it still has a low lignin content compared to wood [35]. Seed fibers are extracted from a variety of plant seeds. Recently, cotton, kapok, and coconut husk are popular seed fibers used in hybrid natural fiber-reinforced polymer composites. A new class of cellulose-based reinforcement material has resulted in the emergence of the development of a seed fiber blend, which is used in thermoset composite production. Straw fibers have been used as reinforcing fillers in a several polymers due to their low water content. They are considered to be relatively stable, with little evidence of microbial respiration in most cases [36]. Thus, straw fibers are excellent hybrid natural fiber polymer composites candidates. Meanwhile, grass fibers are lengthened sclerenchyma cells that are present in different parts of the plant. They are most commonly present in the ground and vascular tissues, as they provide mechanical assistance, but they can also be found in dermal tissues [37]. Currently, grass fibers are similar derivatives to the synthetic fibers used in composite materials and other applications. Various studies have reinforced this fiber in a polymer matrix, making them partially biodegradable green composites. Wood is a fibrous complex biological tissue structure found in woody plants that makes up the inner portion of stems (trunks), branches, and roots. Based on plant taxonomy, there are the two main categories of woody species: hardwoods and softwoods. Wood is a biomaterial with an optimal hierarchical system capable of transferring both internal and external forces, making it perfectly suited as a structural material in a variety of applications [38]. 

## 3. Static Mechanical Testing

Mechanical properties are the physical characteristics that a material displays as forces are applied. These properties are the most critical issue in evaluating the behavior of hybrid natural fiber polymer composites in a number of applications. Mechanical testing of polymer composites involves evaluating mechanical criteria to ensure that the material meets the performance criteria set out by industry standards. Typically, the static and dynamic mechanical properties are used to evaluate the performance of the hybrid natural fiber polymer composites.

Static mechanical testing is a destructive method where static forces are applied to the material in the experiment. This is a good way of modeling where the load applied to the material remains relatively constant over time. Typically, the static mechanical testing can determine a variety of mechanical properties of hybrid natural fiber polymer composites including the tensile strength, elastic modulus, elongation, hardness, and fatigue failure of materials [39]. Tensile strength is the ability of a material to withstand a force that attempted to break it apart, while the deformation of a structural element under flexure is determined by elastic modulus. Tensile testing can also be used to evaluate elongation, which reflects the ability of a material to withstand changes in shape without cracking. Meanwhile, the hardness properties of hybrid natural fiber-reinforced polymer composites can be investigated by applying indentation loads normal to fiber diameter and normal to fiber length [40]. Fatigue testing is needed for composite materials, particularly in high-demand applications such as aerospace and wind power. The load frames in this test must have the high stiffness and outstanding alignment that composite testing requires [41]. However, static stress tests cannot accurately simulate many of the dynamically changing forces. Hence, dynamic testing is used to study how a material responds when a force is suddenly applied [42]. Table 3 summarizes each plant’s fiber characteristics and applications in polymer composites.

Another important analysis to determine the performance of the hybrid composites is bending analysis. The main aim of this evaluation especially for hybrid components is to develop a suitable manufacturing method and examine the quality through both microscopic assessment and mechanical testing [84]. The beam theory is the common method of evaluation widely used for many civil, aerospace, and mechanical engineering applications. The conventional beam theories indicate that the bending analysis of the beam under transverse loading depends on support in addition to the other parameters. These theories assume that support is placed at the center-plane of the beam [85]. The most common, the most classical, and most frequently used beam theory is the Euler–Bernoulli beam theory (EBT), which is commonly known as elementary beam theory [86]. This theory assumes that the plane section remains plane after the deformation and disregards the effect of shear deformation. Another theory used to evaluate the bending analysis of hybrid composites is known as Timoshenko beam theory. This theory is also known as the first-order shear deformation theory (FSDT) and developed based on EBT. The Timoshenko beam theory assumes a constant stress distribution across a beam section. In order to correct the inherent deficiency of Timoshenko beam theory, the shear correction factor is introduced due to constant strain assumption [85,87,88].

Generally, hybrid composites or sandwich-laminated structure selected from a fiber based resin-reinforced material that have a high specific modulus and specific strength, can withstand large bending at normal stress and able to resist shear deformation. The existing analytical models of composite beams are based on classical beam theory, without considering non-classical effects including lateral shear deformation, warping, and three-dimensional strain [89]. The manufactured hybrid composites in structural applications usually involve the joining metal and composites constituent through co-molding techniques, as in the study done by Asaee [84]. The hybrid aluminum alloy sheets-reinforced glass fiber-reinforced plastics (GFRP) layers composite was analyzed via quasi-static three-point bending loads. The authors found that the hybridization enables improvement in strength and energy absorption in a three-point bending test by 130% and 90%, respectively. The improvement corresponded to an increase in the mass-normalized peak load and specific absorbed energy, particularly for beams tested for the first loading configuration. In a similar study, Wang et al. [90] manufactured steel-reinforced carbon fiber-reinforced polymer hybrid beams via a resin transfer molding (RTM) technique. The hybrid beam was analyzed through four-point bending and axial crush tests. The findings showed an increase in performance for hybrid composites compared to steel beams alone, observing 19.3% less real intrusion (largest displacement of the indentor) and 26.5% less specific intrusion after hybridization, but increased the overall weight by 9.6%. Meanwhile, Cerbu [91] studied the theoretical model of beam theory using natural fibers, flax reinforced with glass fibers, and compared them with composites reinforced with only flax fibers. The findings showed the application of bending beam theory to validate the bending beam model proposed through this study. The theoretical values of the equivalent modulus of elasticity (Ex) are found to be near to the value determined experimentally for both flax/epoxy and glass/flax and epoxy hybrid composites. Thus, it is stated that the equivalent moduli of elasticity of bending models presented through this work can be applied in any beam subjected to bending made from laminated composite materials.

## 4. Factors Determine the Mechanical Failure

The mechanical properties of the composites system are the most vital part that need to be focused on to study the capabilities of the structural materials. The materials should have the ability to bear high amounts of loads in different directions. In general, there are three principal parameters that determine the end properties of the resulting hybrid composites. First is the choice of the materials used in the composites, including matrix and filler, which depend on the desired applications. This includes the properties and types of the fibers, whether they are long fibers, short fibers, or powder form. The amount of the fiber present in the composite also plays a major role in determining the overall mechanical performance of the hybrid composites. The aspect ratio of the fiber is very important for influencing the mechanical properties of the hybrid composites [3]. The fiber length must be greater than the critical length to allow a high efficiency of reinforcement in the composites particularly during tensile load to enable it to be transferred into the fiber from the matrix throughout shear at the fiber–matrix interface [92,93]. The choice of polymer matrix also plays a major concern in enhancing the hybrid composites. The polymer matrix affects the mechanical properties of the composites based on its high degree of crystallinity, and it can also provide higher strength and stiffness to the whole composite [94].

The second factor includes the method preparation of the composites, involving the design of the product that needs to meet certain specifications and the condition during the composites fabrication. The product design also must consider other factors: whether the hybrid composites will be used in an open environment or indoors as protection storage needs to be considered. The properties such as weight, mechanical strength, density, recyclability, disposability, water absorption, raw material, and manufacturing cost as well as its compatibility with the current recycling system have to be taken into account and meet the standard of the end-product depending on the intended applications [2]. The stacking sequence of the composites plays a major role in determining the mechanical properties of the composites [3]. This includes the design freedom of composites, which includes the aspects such as the fiber orientation, stacking sequence, and preform types. Features that can be taken into account include (1) unidirectional composites, which are significantly stiffer in the fiber direction than similar composites with fiber with multiple directions, (2) placing 0° plies on the outside of 0/90° laminates to increase the longitudinal flexural stiffness without affecting the tensile stiffness, and (3) the choice between unidirectional plies, textile, or random mates, which can affect the impact behavior [95].

The third and also the most important parameter is the interaction properties between the filler and the matrix. These include the use of treatment upon fiber or the application of a coupling agent with the aim of enhancing the interfacial adhesion between the natural fibers and the matrix, thus improving the overall properties of the hybrid composite [2]. According to Shireesha et al. [3], chemical treatment or surface treatment prior to composite preparation is needed to impart better tensile and flexural properties on the composite. The improvement stated here includes the improvement of fiber dispersion in the polymer matrix, which leads to appropriate interfacial bonding with increased distribution and reduces the formation of voids. The pre-treatment of fibers also reduces the water absorption and imparts better mechanical properties. There are a variety of pre-treatment choices for natural fibers hybrid composites preparation comprised of physical and chemical treatment. The physical treatment includes beating and heating, whilst the chemical treatment that is usually applied includes alkalization, silane treatment, acylation, benzoylation, etc. These pre-treatments are able to reduce the fracture toughness and increase the compressive strength [93]. The interaction between fiber and polymer matrix also influences the failure behavior of the composites. According to the study done by Schneider and Lauke [96], the main reason for the individual failure behavior is the internal heterogeneities in the composites. This characteristic was responsible for initiating the failure such as micro-cracks, defects in the laminated structure, fractured fibers of the specimen preparation, etc.

Another factor that leads to the failure of the hybrid composite is the effect of moisture absorption. According to Mochane et al. [2], moisture absorption is a major restriction to the successful utilization of natural fibers in hybrid composites products beyond non-structural and interior applications. Exterior application of hybrid composites allows them to be exposed to various environmental stresses—for instance, humidity, temperature, or UV radiation. The exposure causes aging, which needs to be studied to evaluate their behavior during their service life. In general, the dimensional stability of the composites and their mechanical performance are strongly influenced by hygro-thermal and hydro-thermal conditions, which are related to relative humidity and immersion conditions, respectively [97]. The ability of natural fibers to absorb moisture relies on the hydrophilic nature of the fibers itself. Natural fiber consists of a large number of hydroxyl groups (–OH) are present in hemicellulose and cellulose. Despite the constituents that lead to moisture absorption, lignin is one of the components in natural fibers that are hydrophobic in nature which contain a low –OH-to-C ratio. The cellulose and hemicellulose, on the other hand, have a large –OH-to-C ratio, which makes them accessible to water when the hydroxyl groups are exposed. However, the accessibility of the cellulose component is limited compared to hemicellulose, since cellulose is semi-crystalline. The crystalline region of the cellulose is inaccessible to water molecules, but the amorphous region of the cellulose enables it to gain access to water molecules [98,99].

**Figure 3:**
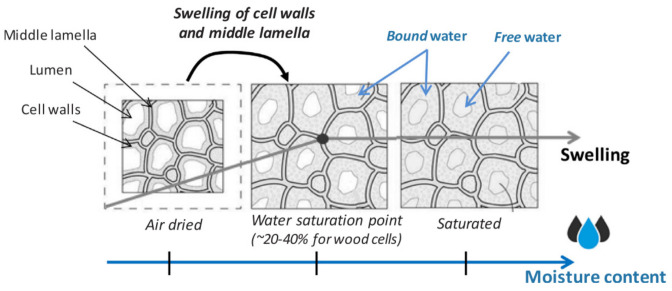
The swelling and water state based on the moisture content in natural fiber cells. Adapted from ref. [97].

The mechanism of water absorption of natural fibers starts with the fibers swelling as the water molecules occupy the space between microfibrils. This phenomenon is known as a temporary microcapillary network. The absorption of water by natural fibers can be in two states: (1) the water bound to the cell walls and middle lamella through the hydrogen bonds with hydroxyl groups (–OH) and (2) free water in micro- and macropores of cell walls, which filled the voids then retained by capillary forces [100,101,102]. The swelling processes of natural fibers are illustrated in Figure 3. As shown in Figure 3, the water vapor was able to penetrate and absorb into the cell walls and middle lamella as the water vapor increases via hydrogen bonds, which leads to significant cell swelling. Consequently, the bound water saturates the cell wall and middle lamella until the water saturation point of plant cells is reached, which is in the vicinity of 20 to 40% for wood cells. The cellular cavities (lumens and porosities) then reached total saturation as it was completely filled by free water.

**Figure 4:**
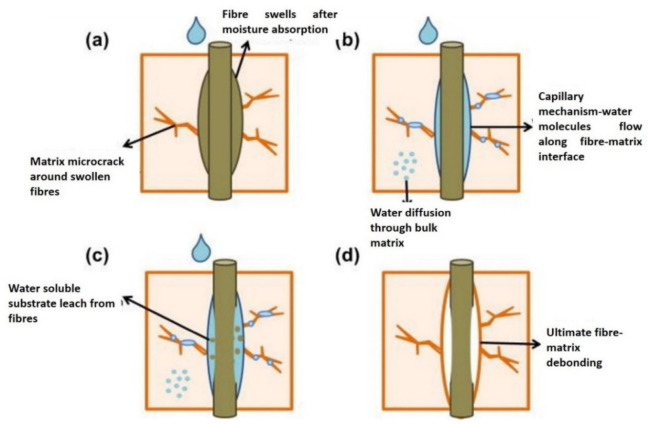
The mechanism of water absorption on the fiber–matrix interface. (**a**) Formation of micro-cracks due to fiber swelling; (**b**) Penetration and water molecules transfer through the micro-cracks; (**c**) Water-soluble substance leaching; and (**d**) De-bonding of the fiber–matrix interface. Reproduced from ref. [2].

Meanwhile, several factors influenced the water absorption properties of the natural fiber hybrid composites including the fiber volume fraction, temperature, fiber nature, difference in water circulation within the composites, degree of crosslinking and crystallinity, diffusivity, and the response between water and the polymer [103]. The water absorption of polymer-based hybrid composites was tested according to the standard of ASTM D570-98. The samples with specific dimensions were usually weighed before and after the immersion of water at specific time points to measure their properties upon absorption of the water. The mechanism composite failure caused by the water absorption on the fiber–matrix interface is depicted in Figure 4. This mechanism is known as diffusion, and it is denoted as a process by which substances are transported from the high concentration area of the system to another area that has a low concentration of water due to random molecular motion. This diffusion mechanism within the composites system involves the following: (1) the direct penetration of water molecules through gaps between polymer chains, (2) water molecules mobility through micro-cracks in the matrix due to the swelling of fibers in the same system, and (3) capillaries bringing the water molecules into flaws and gaps at the interface of the fibers and matrix due to poor impregnation and wetting. As the fiber swells, the penetration of water molecules causes dilapidation, cracks formation, and debonding of the fiber–matrix interface, which leads to the development of osmotic pressure pockets at the surface of the fiber [104]. In addition, the absorbed water molecules act as plasticizers and affect the transfer efficiency, resulting in the deterioration of the mechanical properties.

## 5. Failure Mechanism of Natural Fiber Hybrid Composites

The failure or fracture of polymer-based composites can happen instantaneously and can be disastrous. The composite structure usually includes discontinuities such as cut-outs and holes for joints, which contribute to the critical region of mechanical failure. Therefore, the identification of the factors and mechanism of the failure modes is very crucial and difficult to determine. The failure modes include macro-buckling, crushing of the specimen, shear failure of the specimen, and the swelling of fibers due to moisture absorption, which becomes the main factor of failure mechanism in the natural fiber hybrid composites [93,105].

### 5.1. Crack Propagation and Delamination

Crack propagation in the composite’s structure becomes the main contribution to structure failure. The main cause of the crack propagation is the fracture of the composites, which usually occurs due to the development of displacement discontinuity surfaces within the composites. A fracture is where there is a separation of an object or materials into two more pieces under an action of stress. There are two types of displacement that determine the type of crack, including tensile cracks and shear cracks. Tensile cracks are where the displacement develops perpendicular to the surface of displacement, whereas when the displacement develops tangentially to the surface of displacement, it is known as a shear crack, slip band, or dislocation.

Crack initiation and propagation contribute to the fracture. The manner of how the crack propagates determines the mode of the fracture. There are three different modes of fracture failures (1) Mode I crack—opening mode where a tensile stress is normal to the plane of the crack, (2) Mode II crack—sliding mode where shear stress acts parallel to the plane of the crack and perpendicular to the crack front, and (3) Mode III—tearing mode where a shear stress acts parallel to the plane to the plane of the crack and parallel to the plane of the crack and parallel to the crack front [106]. Bolf et al. [107] elaborate on the crack in the composites based on the opening mode where the crack will usually propagate in the direction of the fiber. However, after the test, the crack will propagate through the matrix regardless of the direction of loading or the angle of the fiber.

In ductile materials, the crack moves slowly, which leads to a large amount of plastic deformation. This type of crack will stop extending unless stress is applied. In contrast, the crack that propagates in brittle materials will continue and grow with increasing magnitude once it is initiated. Crack propagation mechanism within the composite materials is by passes through the grains in the composite’s structure is known as transgranular fracture. Meanwhile, the cracks that propagate along the grain boundaries are known as intergranular fractures [106].

In separate cases, the crack can be developed in composite laminates composed of several layers with different orientation. Natural fibers-reinforced composites, particularly those that have the laminate configuration, experience both intralaminar mechanisms (e.g., matrix cracking, fiber splitting, and fiber/matrix interfacial de-bonding) and interlaminar mechanisms such as delamination. Delamination in particular occurs in an area where the properties of one material are completely different from those of the surrounding material. Delamination is also noticeable as crack propagation that happens between layers of composite materials. According to studies, delamination frequently starts at free edges and/or at discontinuities arising from matrix cracking; in addition, bridging and dynamic effects such as strong interaction between different mechanisms may rise at multiple length scales [107,108].

**Figure 5:**
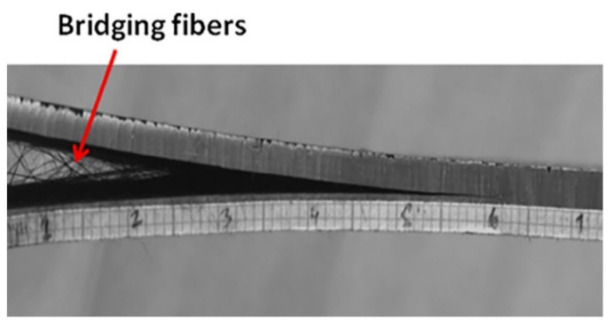
Fiber bridging mechanism of unidirectional laminates. Reproduced from ref. [111].

Delamination in sandwich laminate occurs when the sandwich plates separate from the core with the laminate skins. This occurrence may occur due to one of these factors, including the insufficient core preparation, surface impurities, or premature application of the next layer of laminate as the laminate was subjected to a certain load: for instance, compression or flexion [107,109]. The failure can be caused by stress concentration, low velocity, manufacturing defects, etc. Then, the damage continues to propagate under fatigue loading, which contributes to failure of the composite’s structure. Fiber bridging is an important phenomenon that shields the delamination growth in the composite laminates. The presence of the bridging fibers can resist interlaminar failure [110]. The effect of bridging is more significant in unidirectional laminates than inclined laminates where both upper and lower plies contribute to bridging, as shown in Figure 5. A weak fiber–matrix interface and larger crack tip have been reported as causes of fiber bridging [111].

### 5.2. Fatigue

The fatigue failure also can occur in various types of structural components, which occur below the ultimate tensile strength of the material. It is estimated that 50% of the failure in structural components is due to fatigue failure. This made the fatigue behavior one of the most vital in almost all engineering applications [112]. Fatigue is defined when one material is subjected to repetition cycles of loads, which cause it to lose its required stiffness and strength. Through this process, materials will experience their initial cracks or imperfection especially after a number of cycles and continue to expand and propagate [113]. According to Bolf et al. [107], a composite material subjected to fatigue loads will go through these five features: (1) breakage of the matrix and/or fibers, (2) crevice cracking, matrix fiber peeling, and/or breakage of fiber, (3) delamination, (4) crack growth through delamination and fiber breakage, and (5) breakage of the materials.

The effect of fatigue behavior has been widely studied to understand the development of fatigue behavior in composite materials. To date, there are no reliable methods to perfectly describe the behavior of composite materials based on fibers used and the layouts. Weibull variations of formula are the methods that are often used to accurately predict the method for the damaging behavior of fiber lamina [107,114]. Talreja [115] developed a framework to understand the fatigue behavior of composites based on the configuration of unidirectional and laminates-based composites. As described by Talreja [115], the first region (Region I) extends the scatter band of failure strain, which represents a lack of degradation in strength. Meanwhile, Region II showed the expending of fatigue life from Region I at a certain number of cycles up to the fatigue limit. This region contributes to the progressive mechanism of fiber-bridged matrix cracking. Meanwhile, Region III is the region where no fatigue failure occurs (based on selected large numbers of cycles) lying below the fatigue limit. This study is complemented by the current fatigue failure theories particularly for unidirectional lamina. Dong et al. [116] listed the theories reported by other findings and stated that the first type is the fatigue life theory based on S-N curves or a Goodman-type diagram. Meanwhile, the second type is the phenomenological theory based on residual stiffness and strength models. The third type includes the progressive damage models where local failure mechanisms are considered that apply one or more damage variables related to a measurable manifestation of damage such as transverse matrix cracking or delamination. In separate studies, fatigue failure also occurs in multidirectional laminates. The theories that are used to discuss this phenomenon can be divided into two categories: fatigue failure theories that can be applied to laminates of a specific configuration and fatigue failure theories that can be applied to laminates of any configuration [116].

### 5.3. Microbuckling

Microbuckling refers to the buckling of the fibers involving transverse displacement under compression in the direction of the 3cdevg [117]. The microbuckling of fiber composite laminates initiates at the open hole, and this microbuckle grows from the edge of the hole [118]. This failure mechanism have been studied previously by Soutis [119], who found that the open hole causes a reduction in strength of composite laminates by more than 40% when initiated by fiber microbuckling in the 0° plies at the edge of the hole. The configuration of fibers in a composite’s system plays a major role affecting the compressive properties of the hybrid composites. Chen et al. [120] mentioned that unidirectional fiber-reinforced composites have inferior compressive properties due to the shape instability of the fiber especially at a high aspect ratio. The microbuckling of fibers can occur at a stress level considerably below the strength of the fiber followed by I kinking of fibers, and matrix-tuning is an approach to enhance the compressive strength of unidirectional fiber-reinforced composites.

**Figure 6:**
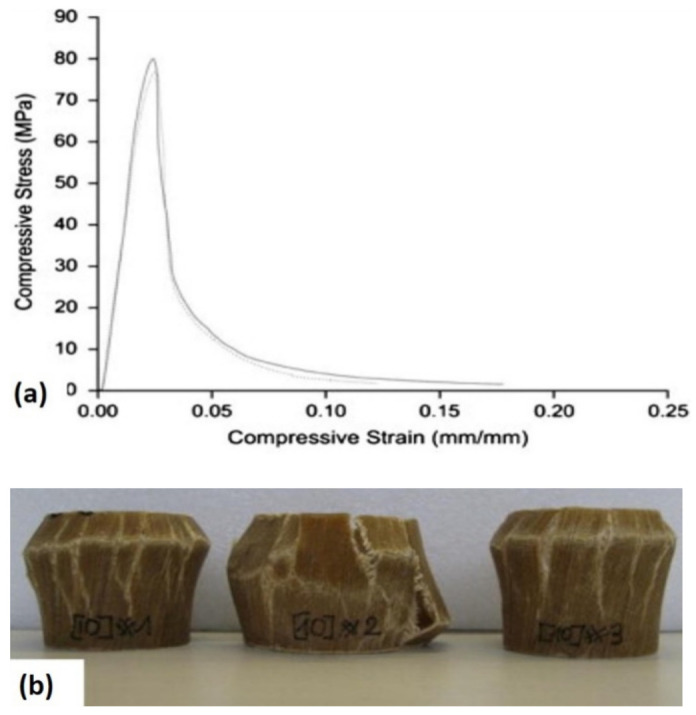
Micro-buckling fracture of a natural fiber composite consisting of hemp yarns with 10° orientation; (**a**) Stress–strain response graph and (**b**) Example of sample fractures in micro-buckling. Adapted from ref. [122].

In separate studies, Tsampas et al. [9] evaluated the effect of monolithic and hybrid multidirectional composites laminates on compressive performance. Through the fractography analysis, the studies showed that the hybridization via multidirectional laminates was greatly influenced by the compressive performance by showing the delamination and interlaminar fracture at the hybrid interface which was more detrimental than that at the monolithic interface [121]. It is important to note that the fracture of natural fiber composites through the microbuckling process is also influenced by the matrix stiffness in shear, which resulted from sensitivity to time, strain rate, and test condition. Schultheisz and Waas [122] also mentioned that the microbuckling can be derived from the initial processing defects of the composites including fiber misalignment or matrix shrinkage due to residual stress and porosity. These factors are very important, as most of the natural fibers may contain multiple technical fibers, which increases the defect content. It is concluded by the author that the microbuckling was affected by the fiber orientation angle, and a similar observation was also found in a previous study related to glass fiber composites. The examples of compressive failure on natural fiber composites in Figure 6 by Wȩcławski et al. [123] show that the fracture was evaluated by a sudden fracture followed by force dissipation due to sliding of the tube walls.

## 6. Hybrid Natural Fiber Polymer Composites

**Figure 7:**
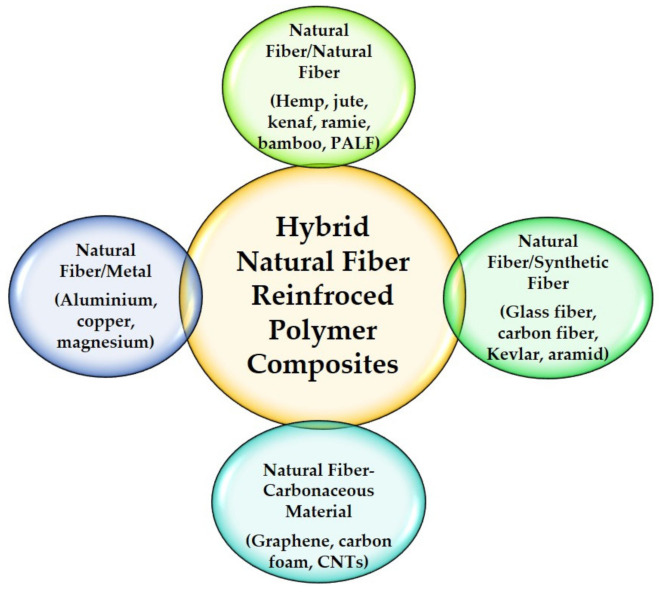
Summary for hybridization of natural fibers with other materials discussed in this section.

**Figure 8:**
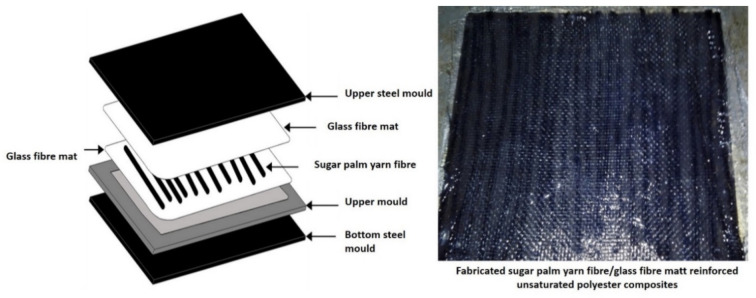
Schematic diagram of the hybridization layer of sugar palm yarn fibers with glass fiber mat-reinforced unsaturated polyester composite. Reproduced from ref. [136].

Natural fiber-reinforced polymer composites have the potential to replace synthetic fibers composites in a variety of applications such as vehicle components, furniture parts, structural construction, and medical devices [124,125,126,127,128]. The hybridization of natural fiber-reinforced polymer composites can be a combination of natural–natural fiber, natural–synthetic fibers, natural fiber with carbonaceous materials, and natural fiber with metal (Figure 7) [124,129]. Hybrid natural fiber composite materials are become challenging to manufacture due to their different characteristics and consideration of the interfacial adhesion for each. The composites processing involves in the forms of simple mixing and sandwich laminates that are lattice and segmented [130,131]. According to Caseri [132], the interaction could be either weak because of van der Waals forces, hydrogen bonding, and weak electrostatic interactions, or it might be strong due to the chemical interactions between the different components. A number of studies on hybrid natural fiber composites have been conducted, and they provide a range of properties that are not possible to achieve with a single type of reinforcement [133,134]. As a result, a balance of mechanical performance and cost reduction for engineering applications could be realized. However, hybrid natural fiber composites are generally limited up to 50% of fiber loading [135]. Figure 8 shows an example of the hybridization layer between sugar palm yarn fiber with glass fiber mat-reinforced unsaturated polyester composites conducted by Nurazzi et al. [136].

### 6.1. Mechanical Performance of Hybrid Natural Fiber/Natural Fiber Polymer Composites

A combination of several types of natural fibers in different forms (e.g., woven, non-woven, long fiber, short fiber, and powder) in a polymer matrix is possible for the development of hybrid composites for a variety of applications such as automotive, aerospace, furniture, etc. [133,137,138,139]. Asim et al. [140] studied the influence of hybridization of PALF and kenaf fiber at different fiber loading on the mechanical properties of phenol formaldehyde composites. It was found that the tensile, flexural, and impact properties of hybrid composites increased with the incorporation of PALF and kenaf fiber, with increasing of kenaf fiber loading. The hybrid composites displayed improved tensile and flexural strengths after adding kenaf fiber up to 70% by weight, due to the fact that the fibers are capable of transporting load; thus, the stress from the matrix to the fiber loading is transmitted. Composites with a higher kenaf fiber content of 35 wt % displayed the highest tensile strength (46.96 MPa) and modulus (6.84 GPa), flexural strength (84.21 MPa) and modulus (5.81 GPa), as well as impact strength (5.39 kJ/m^2^).

The SEM images of the tensile fractured surfaces of hybrid composites revealed that the fibers seem to be evenly dispersed, with good fiber distribution and interfacial bonding between the fibers and the matrix, which has shown the kenaf fiber to have a very strong phenolic resin compatibility. The inclusion of kenaf fiber was found to aid in the effective transition of load from the matrix to the fibers; in addition, kenaf fiber’s mechanical properties are superior in nature to those of PALF. On the other side, the higher aspect ratio of PALF was found to contribute to the increments of impact strength, energy absorption percentage, and flexural modulus of the hybrid composite. A higher aspect ratio and cellulose content of PALF will offer strength in a composite, which can be influenced by a greater breaking energy and higher impact behavior.

Several researchers have determined the mechanical properties of hybrid natural fiber/natural fiber-reinforced polymer composites in woven form. Khan et al. [138] studied the influence of a woven kenaf (K) and woven jute (J) fiber stacking sequence on the mechanical properties of epoxy composites. Two types of three-layer hybrid composites were produced: J/K/J (woven kenaf at the middle part), and K/J/K (woven jute at the middle part). It was found that the maximum improvement in the tensile and flexural properties was in the K/J/K composite, with woven kenaf placed as the outer layers. The tensile strength values of the K/J/K composites have the highest tensile strength of 43.21 MPa compared to J/K/J, which has 40.66 MPa. The strong tensile strength of the woven kenaf fibers on the outside skin means they are able to cope with the tensile stress, while the jute core is pressure-bearing and consistently distributed in hybrid composites. The use of high-strength fibers in the skin layers was thought to result in the best mechanical characteristics.

Other researchers found similar findings in hybrid composites, where when more resistance material is employed as skin, which is the main carrying component in the assessment of tensile strength, the tensile properties become higher [141]. In the case of flexural properties, using woven kenaf as the outer layer significantly increased the flexural strength to 75.57 MPa in the K/J/K hybrid composites, which demonstrated that the K/J/K configurations had a stronger interfacial bonding characteristic, resulting in improved flexural efficiency. In addition, the fiber stacking pattern also improved the flexural characteristics, because the fibers may have carried the flexural-applied stress, which improved the hybrid composite’s toughness and bending strength. The SEM analysis revealed that the K/J/K composite had better structural properties, with fewer fiber breakages and less fiber pull-out.

The hybridization of a plain woven kenaf (K) and plain-woven sisal (S) fiber-reinforced bio-epoxy composite as a result of stacking sequences was carried out by Yorseng et al. [142]. In this study, kenaf/sisal fiber fabrics-reinforced bio-epoxy composites were laminated into several sequences—namely, KKK, SSS, KSK, and SKS—and their mechanical properties under accelerated weathering conditions were evaluated. Compared to hybrid composites, the neat bio-epoxy exhibited higher tensile strength, elongation at break, and impact strength before the accelerated weathering test, which indicated that the applied stress is not efficiently transmitted from the matrix to the kenaf or to the sisal fiber fabrics. However, when samples were exposed to weathering conditions, the tensile modulus of reinforced hybrid composites (KKK, KSK, KSK, SSS) showed better weathering resistance and more mild mechanical properties than the neat bio-epoxy matrix. Additionally, it was found that the composites of KSK and SKS demonstrate a synergetic effect on the tensile strength compared with the composites KKK and SSS. This is due to the positive hybridization that affect in improving the properties of the polymer composites. The findings also showed that the neat bio-epoxy is vulnerable to degradation, where weathering causes the bio-epoxy samples to degrade, and the mechanical properties decreased. Thus, they concluded that hybrid composites strengthened with kenaf and sisal fibers will keep their properties and suggested the use of natural fiber plant waste materials to produce a semi-structural composite.

Ismail et al. [143] studied the properties of hybrid composites made from a kenaf (K) and bamboo (B) mat epoxy composite using the hand lay-up method at 40% of fiber loading. Three different fiber ratios were used, namely 70B:30K, 50B:50K, and 30B:70K. According to a study, the 50B:50K of the fiber ratio in hybrid composites resulted in the highest impact strength of 45 J/m, compared to the only kenaf-reinforced epoxy composite and the 30:70 fiber ratio in bamboo–kenaf hybrid composites, which had impact strengths of 40.6 and 37.8 J/m, respectively. The strength increase was due to the geometry of the woven kenaf structure, which offers excellent impact strength. Additionally, the excellent results of flexural and impact strength of hybrid composites were due to the strong interfacial bonding between the fibers and the matrix. The SEM images of the flexural fractured surface of the hybrid composites showed that there was less fiber pull-out and fewer fiber breakages and voids compared to other composites. This indicated a high degree of interfacial bonding between the fiber and the matrix. For impact properties, a hybrid with the ratio of 50B:50K also produced the highest impact strength of 44.8 J/m compared to another composite. The hybridization of kenaf and bamboo fibers was found to increase the synergistic effect compared to non-hybrid composites.

Radzi et al. [144] fabricated the hybrid composites of roselle fiber (RF) and sugar palm fibers (SPF) with polyurethane (PU) resin as per the fiber ratios of 100RF, 70RF:30SPF, 50RF:50SPF, 30RF:70SPF, and 100SPF. The study assesses that the hybridization by melt mixing and hot compression of both natural fibers can improve the impact strength, but not in tensile and flexural properties. The presence of a higher amount of SPF in the 30RF:70SPF sample was found to increase the tensile strength value up to 32.4 kJ/m^2^ compared to the 70RF:30SPF and 50RF:50SPF samples, which had strength values of 30 kJ/m^2^ and 30.2 kJ/m^2^, respectively. The increment was believed to be contributed by SPF having good stiffness and rigidity properties; besides, this type of hybrid composite absorbs impact energy more efficiently than other composite types. They also concluded that increasing the composite fiber content enhanced the mechanical characteristics. Composites with a fiber content of 40 wt % exhibited the best tensile and flexural characteristics, but increasing the fiber percentage deteriorated the properties. However, contradictory results were found regarding the tensile and flexural characteristics, where the composite with 30 wt % achieved better impact properties.

Rahman et al. [145] fabricated the hybrid composites by using alkaline-treated kenaf/jute with polyethylene resin. There were several types of composites, namely of JKPEC (jute/kenaf/polyethylene composites), JKPEPOFAC (jute/kenaf/polyethylene palm oil fuel ash composites), and JKPEPVAC (jute/kenaf/polyethylene/polyvinyl alcohol composites), with treated and untreated kenaf/jute fibers. The tensile results demonstrated that the treated fiber composites have a higher tensile strength and Young’s modulus than the untreated fiber composites. The higher tensile properties were found to be due to the better interfacial bonding of the fiber–matrix, which resulted from the alkaline treatment. The chemical agents decreased the hydrophilic –OH group of the jute/kenaf fiber, resulting in improved interfacial bonding between the fiber and the polyethylene matrix in the composites. Interestingly, the addition of palm oil fuel ash (POFA) and PVA in the panels was found to increase the interfacial interaction. Less fiber pull-outs and fewer impurities were found in the treated JKPEPOFAC panel, and there was also a smoother surface and less agglomeration in the treated JKPEPVAC panel, as PVA serves as a reinforcement filler, obstructing the fiber-OH group was observed in their SEM image.

**Figure 9:**
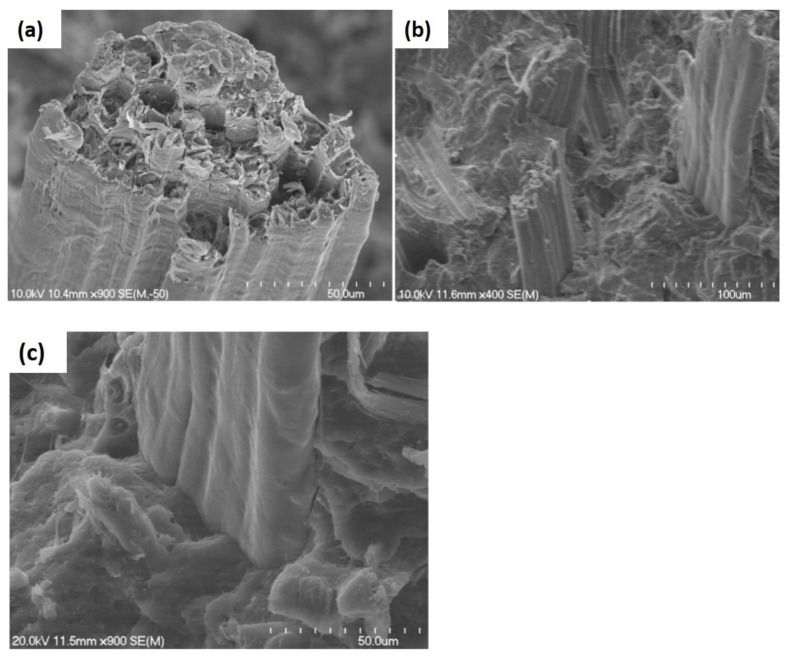
SEM images of hybrid composites: (**a**) fiber fracture, (**b**) fiber pull-out, (**c**) adhesion at the fiber–matrix interface. Reproduced from ref. [146].

Pappu et al. [146] reported the possibility of producing a hybrid composite from hemp and sisal fiber-reinforced PLA composites. In this study, basic fiber properties such as fiber density, diameter, tensile strength, and modulus, in addition to the quality of hybrid composites made from granulated sisal and hemp fibers were evaluated after they were extruded and injection molded using PLA. The results demonstrated a good tensile strength of 46.25 MPa, Young’s modulus of 6.1 GPa, and flexural strength of 94.83 MPa. The introduction of sisal and hemp fibers to composites resulted in a 20% improvement in tensile strength and 43% of improvement was recorded in tensile modulus compared to neat PLA. This improvement was attributed to several factors including the properties of both fibers, where hemp fiber was discovered to have a rougher surface than sisal fiber, implying that it would have stronger interfacial bonding with the matrix. The SEM microstructure in Figure 9 shows improved interfacial adhesion between the PLA matrix and the natural fiber, resulting in improved mechanical and thermal properties of the composites. The studies on natural/natural fiber hybrid composites utilizing both polymers are tabulated in Table 4.

### 6.2. Mechanical Performance of Hybrid Natural Fiber/Synthetic Fiber-Reinforced Polymer Composites

**Figure 10:**
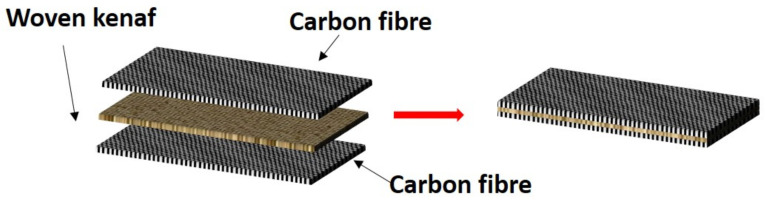
Schematic diagram of hybridization of a woven kenaf with synthetic fibers mat-reinforced polymer composite. Reproduced from ref. [159].

Hybrid composites have been developed by various researchers, combining fibers with epoxy, unsaturated polyester, phenolic, vinyl ester, and thermosetting type polyurethane resins. When, two or more fibers are used for making composites, they are called hybrid composites, for example, carbon fiber/glass fiber, glass fiber/Kevlar hybrid, etc. These type of combination gives an advantage of good strength at lower cost, which can be used for applications that were not possible by using the pure composite. So, the hybridization of composite fibrous material is the key to designing new components having good strength at relatively lower cost [157,158]. Figure 10 shows examples of the hybridization of a woven kenaf with carbon fibers mat-reinforced epoxy composite conducted by Aisyah et al. [159].

Khalil et al. [160] reported on the mechanical properties of EFB/glass hybrid reinforced unsaturated polyester composites. Different ratios of glass and EFB fibers—3:7, 5:5, 7:3, and 9:1—were prepared using resin transfer molding (RTM) with a thickness of 1 mm and a pressure of 5 bar. As a result of the great dispersion of the fiber and the efficient load transmission mechanism of this composition, the mechanical study indicates that the composite with 35% fiber loading exhibited the best value mechanical performance. The inclusion of glass fiber, rather than EFB, was thought to contribute to the higher strength and modulus values. The ratio of natural and synthetic fibers had a significant impact on the composite characteristics, according to the findings. The composites with the highest glass fiber content had the best overall mechanical properties. With increasing EFB concentration, the elongation-at-break increased in a different way. This is owing to the fact that EFB had a high strain-to-failure range of 8–18%, while glass fiber had a strain-to-failure range of 3%.

A comparison of a mix of natural jute and glass fibers with a manufactured glass fiber composite for vehicle bumper materials was conducted by Olorunishola and Adubi [161]. The structure of the material, as well as its hardness and impact characteristics, were investigated in the bumper beam material analysis. Using the hand layup method, three samples of hybrid, namely Cs (30% jute, 10% glass fiber), PNFC (40% natural twisted jute fiber), and GF-C (40% glass fiber) in a PP matrix were created, and their properties were evaluated. The findings showed that the hybrid Cs composite has a superior hardness strength of 65.5 HRB and an impact strength of 11.61 J compared to the others, which indicated that the hybrid Cs composite may eventually replace commercial GF-C in automotive structural applications such as bumper beams.

Petrucci et al. [162] explored vacuum-infused hybrid composite laminates of basalt, glass, flax, and hemp reinforced with epoxy resin with a fiber volume fraction of 21 to 23%. Their mechanical properties such as tensile, bending, and interlaminar shear strength were examined, and they found that the hybrid laminates were found to be better than those of the hemp and flax fiber-reinforced laminates but lower than those of the basalt fiber laminates. Interestingly, the hybrid glass and flax with basalt laminates had the best performance compared to other composites due to hemp fibers having a higher tendency to fibrillate, as well as an intrinsic irregularity in their textile structure. In addition, flax, hemp, and basalt laminate demonstrated the best energy for penetration among the hybrids, which was perhaps due to efficient mechanical locking between the layers [163].

Flax fiber composites have been found to exhibit suitable mechanical properties for general applications, where the flax and carbon fiber hybrid composite reached a maximum strength with only 20% of flax fiber volumes [164]. The study on the mechanical properties of waste sisal/glass, sisal/carbon hybrid fiber-reinforced PP composites shows that increasing the sisal fiber weight content in the composites increases the coefficient of friction. The abrasion volume of sisal/glass hybrid composites was shown to be significantly lower than those of sisal/carbon hybrid composites for the same hybrid ratios. The 4.068 GPa tensile modulus of sisal/carbon composites was significantly higher than that that of sisal/glass hybrid composites with 3.136 GPa [165]. Major factors that affect the mechanical properties of fiber-reinforced polymer composites are the fiber length, fiber wt %, volume fraction (Vf)%, and fiber orientation. In general, sisal fibers are used in diverse forms such as unidirectional, randomly oriented short fibers, weave (plain, twill, and mat forms), and different orientations (0-, 90-, and 45-degree plies, etc.) [166]. The strength properties of natural fiber composites are somewhat lower, because they are less stiff and typically less brittle. Reinforcing glass fiber into the sisal PP composites enhanced tensile and flexural properties without any effect on the tensile and flexural module. In addition to this, adding sisal fiber with glass fiber improves the thermal properties and water resistance of the hybrid composites. These results were also reported by previous researchers who studied sisal glass hybrids with silica [167].

Vinayagamoorthy et al. [168] studied the effect of hybridization composition and chemical treatment on natural and synthetic fiber composites. Woven jute/vetiver/glass and vinyl ester resin were prepared by using hand lay-up techniques. Experiments on flexural behavior showed that natural fibers may replace 15% of glass fibers without compromising composite characteristics. The composite that contained 17% vetiver and jute fibers had the highest flexural strength among the natural fiber hybrid composites, while the composite that contained 10% of vetiver and jute fibers with an addition of glass fiber had the highest flexural strength among the natural–synthetic fiber hybrid composites. The results indicate that hybrid composites have intermediate mechanical properties compared with those of jute and glass composites. Increases in glass content enhance the tensile strength by up to 15%; however, too much glass fiber inclusion is not recommended for attaining optimum tensile characteristics. The mechanical result can be increased by modification techniques of the natural fiber to increase the compatibility and stiffness of natural fiber [169].

Islam et al. [170] studied the effect of heat treatment on the properties of recycled polypropylene (RPP), oil palm empty fruit bunch (EFB), and/or glass fiber (GF) using extrusion and injection molding techniques. The tensile strength for a hybrid RPP/EFB/GF increased by 31.1 MPa and its tensile modulus is 1.4 GPa. Meanwhile, for flexural testing, the flexural strength increases by 33.3 MPa, and the flexural strength is 1248 MPa. The hybridization of the EFB with GF increased the tensile strength, Young’s modulus, elongation at break, and the impact strength of the hybrid composites with the addition of GF content [171]. This may be owing to the polymer’s inclusion of a stronger GF. They also found that heat treatment improves the tensile characteristics, demonstrating the efficacy of the combined impact of temperature and treatment time. However, at severe treatment conditions (90 °C and 120 min), the composite properties reduced due to cellulose fiber breakdown as a result of overtreatment [171].

A hybrid composite with superior mechanical properties is produced, which is suitable for low-cost applications when glass or carbon fibers are incorporated along with natural fiber in the composites system [172]. As a result of the presence of high strength jute fibers in their intermediate layers and good adhesion with matrix after the alkaline treatment, hybrid areca sheath–jute–glass composites demonstrated good tensile, flexural compression, and shear behavior, followed by a pure jute fibers–glass fabrics composite. It was also noted that due to the greater lignin concentration of areca sheath fibers compared to jute fibers, composites with a low amount of areca sheath fibers exhibited very little elongation when compared to the other composites. The cellulose content in fibers is essential in determining tensile strength; the greater the cellulose concentration, the higher the tensile strength, owing to a higher degree of polymerization with matrix and resistance to tension.

Nurazzi et al. [136] investigated the hybridization of sugar palm yarn/glass fiber reinforced unsaturated polyester hybrid composites. The highest tensile strength, tensile modulus, flexural strength, and flexural modulus were achieved at 40 wt % of fibers reinforcement with a ratio of 50:50 wt % of sugar palm yarn fiber and glass fiber-reinforced unsaturated polyester composites. The increase of glass fiber loading had a synergistic effect on the mechanical properties to the composite’s structure due to its superior strength and modulus. This is also attributed to an effective layering design of balance (50:50) between the outer layer of the skin of the composite glass fiber mat, and the sugar palm yarn fiber acts as a core for the composite structure. A similar observation was observed by Sapuan et al. [173], who studied longitudinal basalt/woven glass fiber-reinforced unsaturated polyester–resin hybrid composites. The incorporation of basalt and glass fiber would enhance the tensile strength of the composite. This is because basalt fiber has higher strain, strength, and modulus than glass fiber [174].

Md Shah et al. [78] investigated the influence of powder bamboo and glass fibers on the mechanical properties of a thermoset polymer, Epoxamite 100. A sandwich-structured hybrid composite was fabricated with a layer of woven E-glass fiber embedded each at the top and bottom layer of bamboo-filled epoxy. The purpose of the incorporation of woven glass fiber was intended to slow down the crack propagation in composites. It was found that the lowest bamboo powder loading of 10% marked the highest tensile strength, where the strength decreased as the loading increased. Raghavendra Rao et al. [175] studied the flexural properties of bamboo/glass fiber-reinforced epoxy hybrid composites. It was observed that both the flexural properties increased with glass fiber content up to 200 MPa and modulus 8.3 GPa. Retnam et al. [176] studied the effects of fiber orientation (0°/90° and ±45°) on the mechanical properties of hybrid bamboo/glass fiber-reinforced unsaturated polyester composites. Among all the combinations, the hybrid bamboo/glass fiber composites with ±45° orientation exhibited high tensile strength when compared with other combinations due to the load applied not aligning with the fiber orientation for the fast fracture. In addition, it was observed that hybrid fiber shows more densified and compact structure than the non-hybrid of pure bamboo fiber composite. Thus, this leads to better energy and load dissipation toward the composite’s structure. Table 5 shows the summary of works on hybrid natural fiber with synthetic fiber-reinforced polymer composites.

### 6.3. Mechanical Performance of Hybrid Natural Fiber/Metal-Reinforced Polymer Composites

Composites are classified into three categories: metal matrix composites (MMCs), ceramic matrix composites (CMCs), and polymer matrix composites (PMCs). MMCs were found to have benefits such as high thermal behavior, great strength, modulus, and good fire resistance at raised temperatures. As a consequence of these characteristics, MMCs are being used for a broad range of applications. The most widely used metals in the MMCs are titanium, tungsten, boric, and molybdenum. Currently, metal-filled polymer composites have been widely used due to their good electrical and thermal conductivity. In the production of hybrid natural fiber/metal-reinforced polymer composites, fiber metal laminates (FMLs) are usually used. FMLs are lightweight structural materials made up of alternating thin metal (0.3 to 0.5 mm in thickness) and fused to thin composite plies with metal as external surfaces [180]. This mixture produces a laminate material that is thinner, stronger, and more fatigue resistant, as well as having greater impact strength and damage resistance [181]. The most common used FML are aramid fibers and aluminum (ARALL), aluminum and organic fibers (ALOR), glass fibers and aluminum (GLARE and SIAL) and titanium and graphite fibers (TIGR). Various studies on the hybridization of natural fiber with FML have been reported, as listed in Table 6.

**Figure 11:**
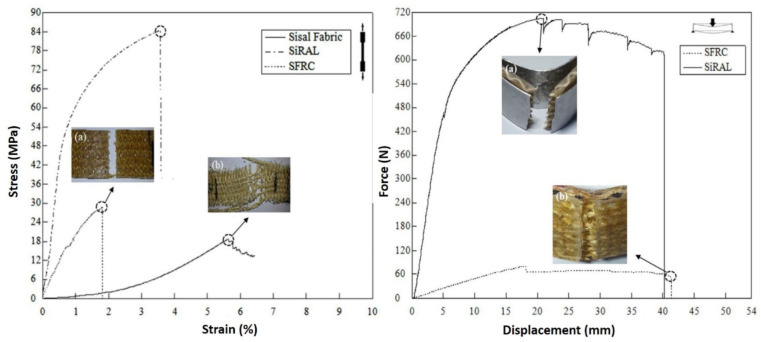
The (**a**) Tensile stress–strain curve and failure mode and (**b**) Flexural behavior and failure mode of sisal fabric, SFRC and SiRAL. Reproduced from [182].

Vieira et al. [182] performed the investigation on composites of sisal fiber-reinforced aluminum laminates (SiRALs) and sisal fiber-reinforced composites (SFRC) on their physical and mechanical properties. Characterizations of the composites showed remarkable enhancements of the tensile, flexural, and impact properties (Figure 11) in SiRAL due to presence of aluminum layers in the composite system. The aluminum layers in FMLs play an important role in composite yielding at high loads, better energy absorption, stable upon deformation, high residual strength, and resistance to short cracks [188]. In comparison with the SFRC, the tensile strength and modulus of SIRALs increased by 132% and 267%, respectively. This behavior may be explained by the fact that under tensile loading, the whole specimen is exposed to continuous tension, while in flexure, only a small portion of the specimen is subjected to the maximum stress [189]. Additionally, aside from the loading volume differential, the thickness of sandwich composites has a major impact on bending behavior due to the area moment of inertia. In addition, the average flexural strength and modulus of SiRALs were considerably higher than those of SFRCs, showing 430% and 973% increments, respectively. However, for SiRALs subjected to impact test, a delamination fracture mode was observed, indicating a poor interfacial adhesion in these laminates.

Chandrasekar et al. [183] studied the tensile and fatigue properties of hybrid FML with the carbon prepreg, flax, and sugar palm fibers epoxy composite prepared by hand lay-up and hot press technique. The effect of different stacking sequences of natural fiber-reinforced composite at the middle part was evaluated. It was concluded that the stacking sequence by different fibers can provide a good balance between the properties. The results indicated that laminate A (flax-based FML) had higher tensile strength, modulus, and toughness than laminate B (sugar palm-based FML). In hybrid composites, the use of flax fibers in the skin layer with sugar palm fiber in the center was found to improve the tensile properties significantly, due to the unidirectional flax fiber’s high tensile strength and stiffness. According to Cai and Jin [190], the load is carried efficiently by unidirectional fibers in the direction of loading. Additionally, it was also highlighted that the benefit of incorporating flax and sugar palm fibers was the improved tensile strength and modulus in comparison to individual flax and sugar palm-based FML, where the hybrid panel demonstrated intermediate tensile properties. In terms of fatigue properties, flax-based FML had a longer fatigue life in the range of 104 cycles and failed with the standard fiber bridging impact. Meanwhile the hybrid composites (laminate C and D) showed significantly longer fatigue life than the sugar palm-based composite.

**Figure 12:**
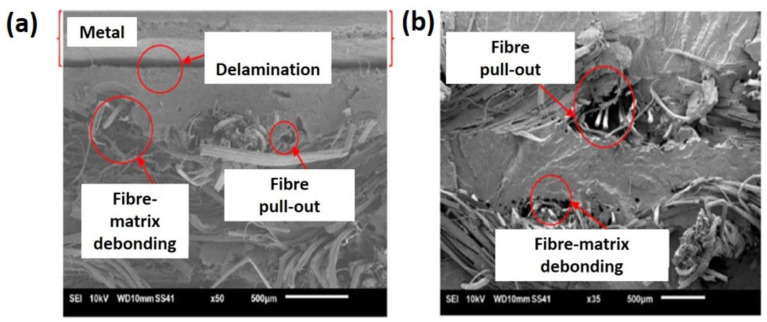
SEM micrograph of tensile fracture of (**a**) NFML and (**b**) NF composite. Reproduced from ref. [191].

Ishak et al. [191] prepared natural fiber FML polymer composite that consisted of two layers of kenaf woven fabric reinforced with PP matrix and aluminum sheets as outer layers. In this work, the effect of water absorption on the tensile properties of natural fiber metal laminate (NFML) and natural fiber composites were studied. The results indicated that the presence of water did not affect the performance of tensile properties of the NFML composite, where NFML absorbed only 0.67% water and had 0.51% thickness swelling compared to the natural fiber composite, which absorbed about 1.95% water with 7.91% thickness swelling. This is due to the outer skin of the aluminum sheet that created a barrier against water absorption, with just part of the vulnerable edge absorbing water. This showed that the NFML polymer composite is long-lasting and has a lower effect on humidity degradation. The tensile strength and Young’s modulus values of the NFML composite are 95.52 MPa and 18.13 GPa, while the values of the natural fiber composite are 35.83 MPa and 6.07 GPa, respectively. After water absorption, the tensile strength of the NFML composite and natural fiber composite decreased by 0.15% and 13.54%. This is because the water content creates voids at the fiber/matrix interface, reducing load transition from the matrix to fiber and lowering the tensile properties. The SEM images in Figure 12 revealed that the NFML’s aluminum begins to delaminate as a result of the load being transferred to the aluminum and natural fiber composite, while the natural fiber composite has a high fiber debonding matrix and a fiber pull-out with poor tensile strength properties, as seen in Figure 12. This is attributed to a lack of adhesion between the fiber and the natural fiber composite matrix, which results in a reduction in load-carrying capacity.

Sivakumar et al. [192] prepared hybrid plain weave kenaf fabric with annealed aluminum laminates reinforced and PP as a matrix material using the hot press compression method. The effects of kenaf fabric layers (one, two, and three layers), fiber orientations (0°/90° and ±45°), and chemical treatment (untreated and alkaline treated using sodium hydroxide (NaOH)) on the tensile properties were investigated. It was found that utilizing one layer of kenaf fabric in untreated FMLs produced the maximum tensile properties as opposed to FMLs with two and three layers of kenaf fabric. However, when two layers of kenaf fabric were added to the laminates, treated FMLs demonstrated the strongest tensile properties. The porosity of natural fiber-reinforced polymer composites continues to rise as the fiber content increases and will affect the stress concentration distribution when load is applied. As a result, increasing the fiber content decreases the tensile properties of this material. For fiber orientation, the tensile properties of FMLs with 0°/90° are superior to those of FMLs with ±45° due to the higher stress uptake capacity, which enables stress to be transferred uniformly through the fibers. The higher fiber loading capacity in this direction allows uniform stress transfer along the fibers at principle and transverse directions, thereby improving the tensile properties. The shear stress created in the ±45° fiber orientation during tensile loading causes a higher stress concentration and deformation at the fiber matrix interfaces, thus reducing tensile efficiency. The scissoring action of woven kenaf strands at ±45° fiber orientation may account for this. Higher elongation is possible due to the lateral contraction of woven kenaf fibers at an off-axis angle until the maximum locking angle is achieved. In the case of fiber treatment, treated FMLs showed greater tensile efficiency than untreated FMLs because of the improvement in the fiber matrix–interface bonding, which affects the tensile efficiency. The NaOH enhances the fiber aspect ratio and the interfacial fiber–matrix area, which makes stress transfer more effective.

### 6.4. Mechanical Performance of Hybrid Natural Fiber/Carbonaceous Material-Reinforced Polymer Composites

Carbonaceous materials come in a broad range of types, including graphene, carbon foam, carbon nanotubes (CNTs), and its derivatives such as single-walled carbon nanotubes (SWCNTs) and multi-walled carbon nanotubes (MWCNTs), and carbon matrix composites (CAMCs), which have a number of favorable properties that make them an excellent source for a range of applications. As opposed to metals and alloys, carbon has a very low density, making it ideal for small and lightweight applications, for example, components for the aerospace industry, automotive, sporting goods, sensors, and electronic components [193,194,195]. Carbonaceous materials such as graphene and CNTs exhibit superior physical, chemical, thermal, and electrical properties [196]. As a result, they seem to be promising filler for improving the performance of natural fiber polymer composite-based products. Carbonaceous materials provide enormous potential for the advancement of different types of composite materials due to their outstanding thermal conductivity and distinct mechanical properties. Numerous researchers investigated the incorporation of carbonaceous materials either as a filler, coating component, or functionalization component in the fabrication of high-end natural fiber polymer-based composites. Table 7 provides information on hybrid natural fiber/carbonaceous material-reinforced polymer composites utilizing graphene, CNTs, and CAMCs found in the literature.

Sarker et al. [197] evaluated the ultrahigh performance jute fiber-reinforced epoxy composites nano engineered with graphene oxide (GO) and graphene flakes. The physical and chemical treatment of jute fibers greatly enhances the fiber packing for the composites and leads to a new fiber composite with increased mechanical performance. In contrast with composites that use untreated jute fiber composites, they showed significant improvement in mechanical properties, as the tensile strength increased by 110% and the elastic modulus increased by 320%. The major explanations for the improved mechanical characteristics of jute/epoxy composites that were GO treated is because the GO and alkaline-treated fiber provide a strong adhesion. In addition, the presence of oxygen-containing functional groups in GO creates a strong interaction between GO-treated jute fiber with the epoxy, allowing them to bear further load. GO’s oxygen-containing functional groups may form a strong connection with heat alkaline-treated fibers, allowing them to transport greater weight from the matrix. The SEM micrograph images reported that the fiber pull-out and poor interfacial bonding are the most common causes of untreated fiber failure, while the failure mode shifted from fiber pull-out to transverse fracture in GO-treated jute fibers. This suggests improved interfacial bonding, which may contribute to improved composite tensile properties. The image of GO-treated jute epoxy composites at a higher magnification cross-section shows that the primary fibers, which were separated after the alkaline and combing processes, are once again firmly linked to each other to form a robust fiber packing within the composites.

Prasob and Sasikumar [208] evaluated the mechanical properties, namely tensile strength, compressive strength, flexural strength, and interlaminar shear strength (ILSS) of a hybrid composite using jute fiber and reduced graphene oxide (rGO) and zirconium dioxide (ZrO_2_) filler-reinforced epoxy composites. The composite performances were analyzed at various temperature conditions; subzero temperatures, −40 °C, −20 °C, and at room temperature. A significant improvement was found in all composite properties with the decrease in temperature in both rGO and ZrO_2_ composites, which was contributed by matrix contraction. The matrix becomes stronger and stiffer but less ductile at low temperature. The tensile strength of the jute/epoxy composite is affected by matrix failure owing to the start and propagation of microcracks at lower temperatures, and the threshold energy for breaking the matrix/fiber interface rises as the specimen hardens after exposure to liquid nitrogen. For the case of compressive strength, maximum value was increased up to 27.3% at −40 °C because of the improved interfacial bond intensity. Moreover, fibrillation generates a significant quantity of uneven surface area, which contributes to the compression strength of composite materials. The compression test of a jute/epoxy hybrid composite shows that the damage process under compression loading includes kink band development, micro-buckling, and rotation of polymeric chains. The flexural strength also shows an increasing trend at subzero temperatures when compared to room temperature. The ductility of the material rises as the flexural modulus falls, allowing for significant deformation before failure. In addition, the result of dynamic mechanical analysis (DMA) showed that the relaxation, loss, and storage modulus, the damping factor, and Cole–Cole plot of ZrO_2_-filled jute/epoxy composites exhibit better energy dissipation and adhesion compared to rGO hybrid composites. The loss and storage modulus curves follow the same pattern as the glass transition temperature (T_g_) curve, while the damping factor curves display no significant difference regardless of the filler or frequency of measurements. This is due to the fact that ZrO_2_ is a filler medium that has the greatest surface interaction, resulting in stronger bonding with the matrix.

The optimum ratio of GO incorporating into bagasse flour PP composite has been investigated by Chaharmahali et al. [202]. In this study, they developed several types of composites with bagasse flour reinforcement ranging from 15 to 30 wt % and NG from 0.1 to 1.0 wt %. They reported that the mixture of 0.1 wt % of GO and 30 wt % bagasse flour was able to achieve superior tensile and flexural strength values of 30% and 7%, respectively. The improvement of mechanical properties is probably linked to the large aspect ratio, high interfacial contact area of the GO particles, and better stiffness properties of GO. They also reported that adding more GO (more than 0.1 wt %) does not increase the mechanical properties significantly due to nanoparticle agglomeration during the processing of the composite and caused an agglomeration of GO in the composite structures.

Mohan et al. [209] investigated the mechanical properties of hybrid MWCNTs with banana–jute–flax fiber-reinforced composites by changing the stacking sequence of the fiber layers and wt % of MWCNTs. The hybrid composites consist of eight layers of natural fibers, with two different stacking sequences of JBFBFBFJ (jute fiber layers at the skin layers, banana and flax fiber layers are arranged at the core layers) and FBJBJBJF (flax fiber layers at the skin layers, banana and jute fibers at the core layers). The wt % of MWCNTs used are 0.5% and 1%, with epoxy resin as a matrix. They reported that the hybridization of natural fibers incorporated with MWCNTs at different stacking sequences demonstrates a significant influence on mechanical properties. It was found that the composite with a stacking sequence of JBFBFBFJ and 1% MWCNTs shows an improvement in tensile, compressive, and hardness properties compared with FBJBJBJF. The enhancement is easily explainable with the properties of jute fiber, which is more ductile than the flax fiber. However, the impact energy in JBFBFBFJ is reduced while increasing the wt % of MWCNTs up to 1% due to the reinforcing effect of MWCNTs. The explanation is because flax fibers have a higher proportion of elongation and cellulose content than jute fibers.

Thakur et al. [210] varied the percentage of CNTs (1%, 2%, and 3%) using the hand lay-up technique. The results showed that the composites exhibited significantly excellent tensile and flexural properties due to the presence of CNTs. The trend of increment in the tensile and flexural properties with the addition of the CNTs up to 3% in the hybrid composite can be explained by good interactions between epoxy, bamboo fiber, and CNTs. These increases demonstrate that the interface of the bamboo fiber mat has a substantial impact on the properties of the hybrid composites. However, the opposite result was observed in impact properties, where the impact energy was decreased with the increasing amount of CNTs up to 2%. The presence of the hybrid composite makes it brittle. As a result, the material’s impact strength decreases as the proportion of CNTs in it increases, causing it to break under dynamic load.

Chen et al. [211] studied the performance of kenaf fiber and MWCNTs-reinforced PLA composites at different compounds such as fiber treatment (untreated and annealing treatment), MWCNT content (0 and 1%), kenaf fiber content (0, 10, 20, 30, and 40%), and PLA content (59, 69, 79, 89, and 100%). They found out that the use of a silane coupling agent resulted in chemical bonding, which enhanced the crystallinity, mechanical properties, heat resistance, as well as interfacial adhesion, which in turn improved the overall properties of the hybrid composite. When the kenaf fiber content was greater than 20% (annealed), with 1% of MWCNT content shows increasing in tensile strength, which is enough to drive the formation of the trans crystalline structure needed for high strength. The PLA was forced to expand in one direction due to the strong heterogeneous nucleation behavior on the fiber surface, resulting in the creation of a columnar crystalline layer perpendicular to the fiber surface axis. Furthermore, the presence of crystallographic connections between the fiber and polymer crystal structures is shown to be a crucial parameter in determining the characteristics of composites.

Nourbakhsh et al. [212] analyzed the influence of MWCNTs and maleic anhydride grafted polypropylene (MAPP) as a reinforcing agent on the properties of bagasse and poplar fiber-reinforced PP composites. The different MWCNT loading (0 wt %, 1.5 wt %, 2.5 wt %, and 3.5 wt %) and MAPP loading (0 and 3 wt %) were used in this experimental work. The results of this study showed an improved mechanical performance of bagasse stalk/poplar PP composite with high loading of both materials. The composites’ tensile and flexural strengths increased by 20% and 5%, respectively when 2.5 wt % MWCNT was added, which was attributed to several factors including chemical interactions that enhance the fiber–matrix adhesion. The tensile strength of poplar-based composites was higher than that of the bagasse-based composite, as poplar fiber has a high fiber length and aspect ratio. In the case of MWCNT loading, composites containing 2.5 wt % of MWCNT provide the highest mechanical characteristic improvements for both fibers. The results also show that well-dispersed nanoparticles result in improved stress or strain distribution in the composite at 1.5 wt % and 2.5 wt % of MWCNT loading. However, as the MWCNT loading increased up to 3.5 wt %, the impact strength was decreased significantly. A decrease in the impact intensity with increasing MWCNT may be attributed to the increase of MWCNT agglomeration chance, which produces tension regions requiring less energy to prolong the spread of cracks.

## 7. Applications of Hybrid Natural Fiber Polymer Composites

Hybrid natural fiber composites have been widely applied in various structural and engineering applications due to their low production cost, high strength-to-weight ratio, and simple manufacturing process. Moreover, the natural fiber composites also established a good combination of mechanical properties such as impact strength, tensile, bending and compressive behavior, creep, and fatigue properties for structural applications [213,214,215]. However, utilizing hybrid natural fiber polymer composites in structural applications is challenging since various issues and factors need to be addressed in previous sections. These major issues of natural fibers restrict the popularity of hybrid biocomposites in industrial applications, including their high flammability [216], lower water barrier properties [217], inconsistency of raw materials and their properties [218], and the bonding behavior of the cellulosic fiber and matrix [219]. Several factors influence the properties of natural fibers such as the part of plant harvested, the climate during the growing period, and the maturity of the plant [220,221,222]. These factors lead to a higher degree of uncertainty toward the properties of natural fibers for product development.

### 7.1. Aircraft Applications

Aerospace industries and commercial aircraft components have been the highest uses of hybrid natural fiber composites. Unlike other land and water vehicles, aircraft require placing higher attention on safety and weight. In this case, aircraft have to be designed and developed using highly specific properties of materials such as polymer composites. Currently, glass and carbon hybrid composites are implemented in these vehicle components due to the low raw material cost and high mechanical strengths. For instance, hybrid fiber-reinforced epoxy composites would result in enhanced performance of the aircrafts interior panel. The application of flax fiber in hybrid fiber-reinforced epoxy matrix composites shows that the sound absorption coefficient was about 20% higher than the glass fiber counterpart at both low and high frequency levels [223]. In addition to this statement, bamboo could be a potential candidate to implement as a sub-constituent in flax/epoxy composites since bamboo/epoxy has 14% and 9% higher tensile and compressive strength, respectively, as compared to flax/epoxy composites [224]. Hence, flax hybrid natural fibers composites demonstrated potential wideband sound absorption and met the aircraft interior panel criteria.

**Figure 13:**
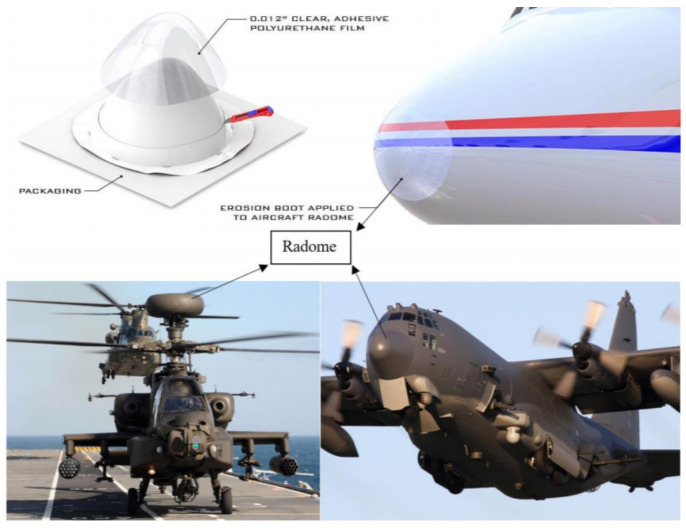
Potential application of hybrid natural fiber composite in aircraft radome.

Moreover, aircraft radome (Figure 13), which shield radar antenna from weather, aerodynamic loads, and bird strike have the potential to benefit from hybrid natural fiber composites. Currently, glass fiber composites are used primarily due to their ability to provide radio-frequency transparency. Radio-frequency transparent materials are materials in which radio frequency fields can penetrate with no heating occurred. In addition, light aircraft radome need to be high toughness composites with a low dielectric constant. Based on the preliminary review of compilation data of natural fiber such as bamboo, banana, kenaf, oil palm, and PALF by Haris et al. [225], hybrid treated kenaf/glass rei-forced epoxy composites can be the potential material to be implemented in radome application. To be specific, the standard mold size for generic radome is ranging from 15 to 20 inches. It can be fabricated either via the vacuum bagging technique or hand lay-up to form a dome-shape laminate of glass/kenaf composites. In general, kenaf fiber displays good overall performance compared to bamboo, banana, oil palm, and PALF.

### 7.2. Marine Applications

**Figure 14:**
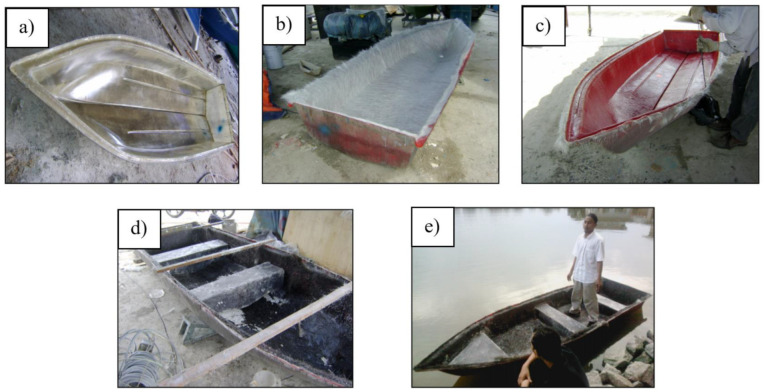
Steps of boat fabrication from hybrid material of sugar palm fiber and glass fiber (**a**) gel coat and catalyst were applied onto the inside surface of the mould (**b**) lay-up process for glass fiber with consistent orientation (**c**) mixture of unsaturated polyester resin and catalyst spread on top of the fiber glass and rolled using iron roller (**d**) lay-up process for sugar palm fiber, followed with the same process at (**c**,**e**) painted with powder reinforcement unsaturated polyester to protect all material such as sugar palm fiber and glass fiber from water. Reproduced from ref. [230].

Generally, marine structures such as ships are usually under constant attack by rust, which leads to defects. The majority of ship hulls are made of carbon steel, which is susceptible to corrosion and has different thermal and electromagnetic detection from long range. These issues have brought material scientists and marine engineers to implement lignocellulosic composites, which are greener than conventional steel. In this case, hybrid configurations of natural fiber composites were used by many researchers due to the limited durability of natural fiber composites (NFCs) materials if subjected to physical–chemical attacks [226]. NFCs tend to have a high water absorption rate, which leads to rapid decreasing of mechanical behavior, since it has weak compatibility between hydrophilic natural fibers and hydrophobic polymer matrices. In this context, the hybridization of natural fibers with synthetic fibers, producing superior aging resistance and better thermal and mechanical stability, has recently attracted attention thanks to their advantages in terms of compromise between environmental impact, mechanical performance, cost, and durability [227,228]. For instance, Calabrese et al. [229] discovered that the flax/glass fibers composite laminate permits improving both the bending strength and modulus by 90% and 128% respectively, even if these properties are lower than those of full glass laminates. The findings demonstrated that the combination of flax and glass fibers-reinforced polymer composites is a practical approach to boost the aging durability especially under marine environmental conditions to replace the conventional steel. Another research study led by Misri et al. [230] evaluates the mechanical performances of a woven glass/sugar palm fibers-reinforced unsaturated polyester hybrid composite. Figure 14 shows the fabrication process of a small boat made out of a hybrid composite from sugar palm fiber and glass fiber.

### 7.3. Civil Construction

**Figure 15:**
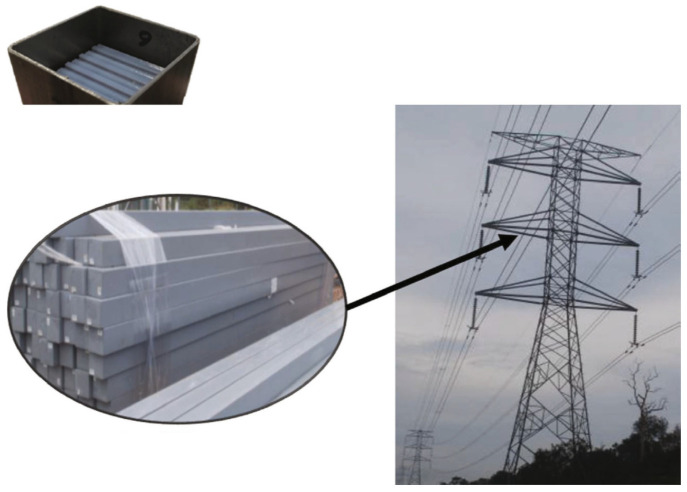
Innovation of honeycomb-filled structure configuration, which potentially can be used in cross-arms.

In civil construction, various research and development teams have implemented hybrid composites in several countries. For example, a hybrid ERP bridge was constructed in Okinawa in 2001 in the form of two span continuous girders pedestrian bridge. Currently, a research team from Malaysia led by Asyraf et al. [220] have suggested the application of hybrid natural fibers reinforced biopolymer composites as cross-arm structure in Malaysian transmission grid system. In the article, they proposed that the composites manufacture using filament wounding technique with inclusion of natural fiber core structure. Figure 15 illustrates the inclusion of honeycomb core in composite tube to allow better strength and stiffness which is potentially use for cross arm application.

### 7.4. Automotive

The most likely methods to meet fuel-efficiency demand with less impact on the environment in the automobile sector is by implementing lightweight materials such as hybrid natural fibers composites in automobile parts. Hybrid biocomposites permit high mechanical strength and stiffness due to the polymeric matrix aiding in transferring the load to the fiber and safeguarding the fiber from the adverse environment and mechanical damage. It is well known that hybrid natural fiber composites exhibit low density, less cost, and are widely available, as car-makers across continents have used them to produce a variety of automotive components. Table 8 displays automotive manufacturers that utilize natural fibers composites in their automotive parts [15,231]. According to Amir et al. [232], it is estimated that around 16 million cars were manufactured in Western Europe per annum, and around 80,000 to 160,000 tons of natural fiber was used.

**Figure 16:**
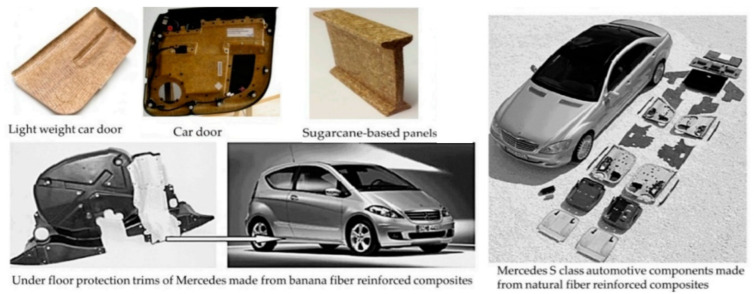
Natural fibers composites as automotive components. Reproduced from ref. [240].

Various research studies have been conducted from cradle to grave on hybrid natural fibers composites in automotive components. Yusof et al. [233] developed a conceptual design for a hybrid palm oil polymer composite automotive crash box. They applied the combination of theory of inventive problem solving (TRIZ), morphological charts, and biomimetics to fulfill the material characteristics, function specifications, force identification, root cause analysis, geometry profile, and design selection criteria [125,234,235,236]. Apart from that, Mansor et al. studied [237] the material selection process for an automotive lever brake using the Analytic Hierarchy Process (AHP). They have chosen kenaf bast fiber to hybridize with glass fiber-reinforced polymer composites for the design of a parking brake component based on the highest overall scores in AHP. Other than that, Adesina et al. [160] reviewed the mechanical properties of hybrid natural fiber composites for a bumper beam. Lower impact properties were discovered based on the mechanical evaluation of the various research studies using hybrid natural fiber as a major limitation in comparison with the conventional glass fiber composites applied as typical bumper beam material. Synthetic fibers aid in compensating for the limitation of natural fibers when used in a hybrid in order to improve the mechanical properties of the polymer composite [238,239]. Figure 16 illustrates examples of automotive components that implement natural fibers composites.

### 7.5. Sporting Goods

In recent times, the sport sectors have grown to become the second most popular entertainment industry. Via technological enhancement, this sector has been reaching worldwide and influencing billions of people. In order to guarantee the safety, security, and sustainability of this sector, cost-effective, efficient, durable, recyclable, and reusable materials were needed by applying advancement in manufacturing and material processes. Thus, the usage of hybrid natural fibers composites seems promising to fulfill the current needs of sport products to bridge the needs of the people and the need for a greener environment. For instance, 90% of field hockey equipment such as sticks, face shields, and helmets are produced by Pakistan using glass/carbon fibers epoxy composites. Due to the high price of synthetic composites of sport goods, this has led researchers and designers to build up sport equipment from hybrid natural fibers composites.

Based on Rashid et al. [241], treated banana/glass fiber biocomposites can withstand higher stresses in comparison with pure glass fiber composites. They discovered the higher flexural strength of hybrid banana/glass fiber biocomposites due to strong bonding between the plies. However, the increasing of banana fiber loading could reduce the strength of composite laminate because the fiber is poor in terms of uniformity, hydrophilic phenomenon, and strength [16,242,243,244]. Thus, it can only be used in hybrid composites at a certain optimized weight percentage. Moreover, the research study also suggested the banana fiber-reinforced polymer matrix along with coir fiber to allow better ductility for the material. Thus, newly designed materials could be a replacement for the man-made fibers (glass/carbon) that are conventionally used in field hockey equipment production [195,245].

## 8. Conclusions and Future Outlooks

The mechanical performance of hybrid natural fiber-reinforced polymer composites has seen a lot of research and development in recent decades. Fiber selection, extraction, handling, and interfacial engineering, as well as composite manufacturing, have all advanced. This paper examined studies on enhancing the strength, stiffness, and impact strength of the hybrids, as well as long and short-term performance. In terms of stiffness and cost, natural fiber polymer composites are now competitive with other synthetic polymer composites; tensile and impact strength values are approaching synthetic values. Natural fiber-reinforced polymer composites-based hybrids are also used in a wide range of structural and outdoor applications, including vehicle exterior underfloor paneling, aircraft components, recreational equipment, and marine structures. Several factors that influence the mechanical failure on the hybrids have been explained; to summarize:

(i) The most common source of crack propagation is composite cracking, which is caused by the formation of displacement discontinuity surfaces within the composites.
(i) Fatigue failure may occur in a variety of structural components that are below the material’s ultimate tensile strength. Fatigue failure is thought to be responsible for half of all structural component failures.
(ii) Microbuckling of fiber composite laminates begins at the open hole and spreads outward from the hole’s tip.

Thus, more research is needed in order to broaden their application spectrum, which includes the following:

(i) Improving moisture resistance and fire retardancy.
(ii) Appropriate concept details can be created in order to popularize the use of these new materials. When hybridization is attempted, more research into the effects of natural fibers on aging is required.
(iii) Since natural fiber-reinforced polymer composites do not provide the expected strength values based on the law of mixtures, comprehensive basic studies on factors related to strength, such as interface bonding and fracture mechanisms, will be conducted to aid the future production of these composites for appropriate applications.

However, several studies have been conducted to determine the impact of natural fiber hybridization at various fiber loadings on their mechanical properties. The introduction of natural fibers hybrids improved the tensile, flexural, and impact properties of hybrid composites, as it increased the fiber loading. The tensile and flexural capacities of the hybrid composites were increased by up to 70% by weight. Morphological analysis of hybrid composite composites showed that the strong fiber propagation and interfacial bonding between the fibers and the matrix improved their mechanical strength. Overall, the growth of hybrid natural fiber polymer composites is at a rapid pace, and their application seems to have a bright future in the years ahead.

## Figures and Tables

**Table 1:** Chemical composition of natural fibers.

Natural Fiber	Lignocellulosic Components (%)			Ref.
	Cellulose	Hemicellulose	Lignin	
Sugar Palm	43.88	7.24	33.24	[21]
Bagasse	32 to 34	19 to 24	25 to 32	[22]
Bamboo	73.83	12.49	10.15	[23]
Flax	60 to 81	14 to 20.6	2.2 to 5	[24]
Hemp	70 to 92	18 to 22	3 to 5	
Jute	51 to 84	12to 20	5 to 13	
Kenaf	44 to 87	22	15 to 19	
Ramie	68 to 76	13 to 15	0.6 to 1	
Sisal	65.8	12	9.9	[25]
Pineapple	66.2	19.5	4.2	[26]
Coir	32 to 43	0.15 to 0.25	40 to 45	[27]

**Table 2:** Comparison the physical and mechanical performance of natural fiber with synthetic fibers.

Fiber	Density (g/cm^3^)	Tensile Strength (MPa)	Elongation at Break (%)	Tensile Modulus (GPa)
Sugar Palm	1.292	156.96	7.98	4.96
Bagasse	1.5	290	-	17
Bamboo	1.25	140 to 230	-	11 to 17
Flax	0.6 to 1.1	345 to 1035	2.7 to 3.2	27.6
Hemp	1.48	690	1.6 to 4	70
Jute	1.3	393 to 773	1.5 to 1.8	26.5
Kenaf	1.45	215.4	1.6	53
Sisal	1.5	511 to 535	2.0 to 2.5	9.4 to 22
Ramie	1.5	560	2.5 to 3.8	24.5
Pineapple	0.8 to 1.6	400 to 627	14.5	1.44
Coir	1.2	138.7	30	4 to 6
E-Glass	2.5	2000 to 3500	0.5	70
S-Glass	2.5	4570	2.8	86
Aramid	1.4	3000 to 3150	3.3 to 3.7	63.0 to 67.0
Kevlar	1.44	3000	2.5 to 3.7	60

**Table 3:** Summary of natural fibers characteristics and applications in polymer composites.

Natural Fiber	Description	Ref.
Bast fiber		
Flax fiber	- Flax (*Linum usitatissimum* L.) is typically grown in temperate climates including France, China, and Belarus. - According to the UN’s Food and Agriculture Organization (FAO), flax was cultivated on a total area of 240,293 hectares (0.24 billion m^2^) in 2018, yielding 868,374 tons of flax. - Flax fibers have been widely utilized as a reinforcement material in a variety of polymer composites, but polypropylene (PP) is the most suitable for flax fibers due to its low density, low thermal expansion, superior water resistance, and potential to be recycled.	[43,44,45]
Hemp	- Hemp (*Cannabis sativa*) is typically grown in Asia and Europe, and this fiber used to make ropes, clothes, and paper due to its high yield and flexibility. - In 2018, 60,657 tons of hemp tow waste, or beaten stalks of the hemp plant, were recorded worldwide - Hemp is undeniably a versatile fiber; however, non-cellulosic ingredients such as hemicelluloses, lignin, pectin, fat, and waxes must be removed before being used. - Hemp fibers have also been used to reinforce polymer composites. Neves et al. [46] used continuous and aligned hemp fibers to reinforce epoxy and unsaturated polyester at 10, 20, and 30 vol %. Epoxy composites outperformed unsaturated polyester composites in terms of flexural and tensile strength when the hemp fibers loading was higher.	[43,46,47,48,49]
Jute	- Jute has reportedly been used to fabricate textiles in the Indus valley civilization since the third millennium B.C. It is the most important fiber in Bangladesh and Eastern India, and it is also recognized as the golden fiber of Bangladesh. - Jute is mainly produced in India, Bangladesh, and China. It was harvested on 1,546,953 ha (1.55 billion m^2^) around the world in 2018, with a total volume of 3,633,550 tons. - It has a high aspect ratio, superior strength-to-weight ratio, and excellent insulating properties. Different chemical treatments may be needed to improve the mechanical properties of the jute fiber. - Gupta et al. [50] stated that jute fiber has been reinforced in a variety of composites including epoxy, unsaturated polyester, vinyl ester, low-density polyethylene, high-density polyethylene, and PP with good mechanical properties.	[50,51]
Kenaf	- Kenaf (*Hibiscus cannabinus* L.), also known as Deccan hemp or Java jute, is an herbaceous plant native to southern Asia. - Kenaf fiber can be extracted from the bark (bast) and core of kenaf stalks, which make up to 40% and 60% of the stalk’s dry weight, respectively. - Kenaf was first introduced to Malaysia in 2010 as a tobacco replacement and has grown drastically to become Malaysia’s third most important industrial crop after palm oil and rubber. - The National Kenaf and Tobacco Board of Malaysia has allocated 2000 ha (2 million m^2^) of land to smallholders for the development of kenaf plantations to meet the growing global demand. - Kenaf has been utilized in a variety of fiber-reinforced polymer composites due to its interesting features: low cost, light weight, renewability, biodegradability, and high specific mechanical properties.	[51,52,53]
Leaf fiber		
Pineapple	- Pineapple (*Ananas comosus*) is extensively planted in tropical areas, and the leaves are used to make pineapple leaf fiber (PALF). - According to Leao et al. [54], a hectare of pineapple plantation could yield about 15 tons of PALF. In 2018, an area of 1,111,372 ha yielded a total of 27,924,287 tons of pineapples (1.11 billion m^2^). In 2019, Malaysia harvested 10,556 ha (10.556 million m^2^) of pineapple and produced 329,365 tons of pineapple. - PALF outperforms other fiber groups as its specific modulus and specific strength are comparable to glass fiber, and its aspect ratio is four times that of jute. - Odusote and Kumar [55] compared PALF-reinforced epoxy composites to glass fiber-reinforced unsaturated polyester composites, revealing that PALF epoxy composites with 40% fiber loading exceed glass fiber-reinforced unsaturated polyester composites in terms of mechanical properties. This suggests that PALF may be a feasible alternative to glass fiber in the production of polymer composites.	[43,54,55,56]
Abaca	- Abaca (*Musa textilis*), also known as Manila hemp, is a non-edible banana species native to the Philippines that is commercially grown in the Philippines, Ecuador, and Costa Rica. - Abaca fibers are extracted from the leaf stems. - The banana can be harvested three times a year, providing a constant supply of abaca fibers. In 2018, 108,131 ton of abaca were produced worldwide. - Abaca has been utilized in a variety of fiber-reinforced polymer composites due to its advantages: long fiber length, strong, flexible, and durable. - Sinha et al. [57] compared the flexural properties of single-layer and double-layer abaca fiber-reinforced epoxy composites and discovered that the double-layer abaca fiber-reinforced epoxy composites had higher flexural properties and could be used for structural applications.	[43,57,58]
Sisal	- Sisal (*Agave sisalana*) is a plant native to southern Mexico that is easy to cultivate and has been grown in many countries outside of Mexico due to its short renewal times. - As a result of its superior strength, sisal has often been used for agricultural twine. In 2018, 198,309 tons of sisal were produced around the world. - Rohit and Dixit [51] stated that sisal fiber has several benefits, including high tenacity and tensile intensity, abrasion resistance, salt water resistance, and acid and alkaline resistance. - Gupta et al. [50] has developed sisal fiber-reinforced epoxy composites with fiber weight fractions of 15%, 20%, 25%, and 30%. As the fiber weight fraction increased, better thermal and mechanical properties were observed.	[43,50,51]
Straw fiber		
Corn	- Corn (*Zea mays*), also known as maize, is commonly grown in many countries. It is plentiful and listed as waste in many countries, including the world’s largest maize producers (Brazil, China, and the United States). - Corn incorporation into polymers could have both economic and environmental benefits. - Corn stover, including the husks, stalks, and leaves, has a high cellulose content and has been shown to be a good source of nanocellulose. - The unique characteristics of corn husk fiber such as flexibility, low density, moderate strength, good elongation, and durability will benefit corn fiber products, including composites.	[59,60,61,62]
Wheat	- Wheat is one of the most widely consumed cereals on the world, and the most common species is bread wheat (*Triticum aestivum*). - The global supply of this fiber is estimated to be around 529 million tons per year. Wheat straw is inexpensive and easy to obtain as agricultural waste. - After harvest, the majority of wheat straw is left to decompose, whereas in certain countries, it is burnt in open fields, posing a threat to air pollution. - Commercial applications of wheat straw are still being studied, regardless of the fact that a limited amount of wheat straw is used as animal feedstock and bedding. - Wheat straw has been discovered to have potential in a variety of applications, including composites, anion exchangers, and panel boards. - Nanocellulose produced from wheat straw using a chemi-mechanical approach had diameters ranging from 10 to 80 nanometers.	[63,64,65,66,67]
Seed/fruit fiber		
Coir	- Coconut fiber (*Cocos nucifera*), also known as coir, is the thickest among all types of natural fiber derived from the inner and outer shell of coconut. - In 2018, global coir demand hit 1,238,725 tons, with Malaysia alone producing 21,521 tons. - Coconut coir fibers have superior mechanical strength and weather tolerance when compared to other fibers due to their higher lignin and microfibrillar angle as well as lower cellulose and hemicellulose content. - Olveira et al. [68] found that a polymer composite containing epoxy matrix-reinforced with 35% coir fiber had the highest flexural modulus.	[43,52,68]
Cotton	- Cotton is an important agricultural crop that belongs to the genus *Gossypium*, subtribe *Hibisceae*, family *Malvaceae*, - Cotton grows around the plant’s seeds that is mainly used to produce clothes and a variety of other daily products for a large portion of the global population. - Cotton has a high cellulose content, which has led to the discovery of nanocellulose synthesis by many researchers. - According to Morais et al. [69], the cotton cellulose nanocrystal (CNC) produced in their study had a diameter of 177 nm, a width of 12 nm, and a 19 aspect ratio. - Composites based on cotton fibers recovered from textiles that make up to 80% by weight fiber have been widely used for thermo-acoustic insulation. - Since cotton is commonly used in fabrics, many attempts have been made to incorporate antimicrobial agents with cotton including argentum/argentum bromide–titanium oxide and copper oxide to enhance the technical quality of fabrics. The findings from Perelshtein et al. [70] and Rana et al. [71] revealed that incorporating these compounds into cotton fabrics resulted in outstanding antibacterial properties, better mechanical properties, selective oil adsorption activity from oil–water mixtures, and ultraviolet-blocking properties.	[69,70,71,72]
Kapok	- Kapok (*Ceiba pentandra*), also known as silk cotton due to its natural luster, is the lightest natural fiber in the world. It is composed of a very thin wall and a wide hollow area filled with air. - The hollowness of kapok circular cross-section cellulose is the highest of all-natural fibers, ranging from 80 to 90%, making it ideal for making low-density polymer composites. - Kapok fiber has a high cellulose content, but it has a lower cellulose content and a higher lignin content as compared to cotton fiber. - Jayaweera et al. [73] found that reinforcing a small amount of nanocellulose extracted from kapok with a diameter thickness of 100 nm and 10 microns significantly improves the mechanical performance of composites.	[73,74,75]
Grass/reed fiber		
Bamboo	- Bamboo has been marketed as a promising future sustainable woody biomass resource owing to its good strength and rapid growth cycle. - Around 1000 bamboo species have been identified in the world, and interestingly, new species are found almost every day. - Bamboo fiber has excellent mechanical properties, rendering it a promising replacement for conventional fibers in composite materials such as glass and carbon. - Bamboo consists of approximately 47% cellulose and 26% lignin, which is much higher lignin content than most natural fibers and other substrates, resulting in its high strength. - Chang et al. [76] produced nanocellulose from bamboo with a diameter of a few tenths of a nanometer, while Han et al. [77] succeeded in producing nanocellulose with a diameter of approximately 15 nm.	[76,77,78]
Sugarcane bagasse	- Sugarcane bagasse is the most widely available lignocellulosic biomass on the world that consists of 41.0 to 55.0 wt % of cellulose, 20.0 to 27.5 wt % of hemicellulose, 18.0 to 26.3 wt % of lignin, and ≈ 7.0 wt % of other compounds such as inorganic materials. - Brazil is the world’s leading sugarcane producer, producing 743.0 million metric tons per year. There are approximately 640 to 660 million tons of sugarcane that could be refined into 28,500 million liters of alcohol, yielding 160 million metric tons of sugarcane bagasse. - Sugarcane bagasse often does not meet the requirements for classification as long fibers. Consequently, the fiber is often left unused, and its use as a composite filler is strongly considered as a potential possibility. - Ghaderi et al. [79] successfully synthesized nanocellulose from sugarcane bagasse with a diameter of 39 nm. - Bagasse fiber has been thoroughly investigated as a polymer reinforcement agent, and there is an excellent potential in developing bagasse-based composites for a range of building, automotive, and construction applications.	[79,80,81,82]
f. Wood fiber		
Softwood	- Softwood trees are non-porous, lack vessels, and grow in a pyramid shape, with a narrow top and a wider bottom with fibers that are longer than hardwood fibers (average length of 4.1 mm and a width of 2.5 μm). - Most of the softwood anatomy is simple, whereby longitudinal tracheid makes up 90% or more of its volume that transports water and provides mechanical strength to the wood. - Since softwood has a higher aspect ratio than hardwood, it is favored for composite applications. Softwood composites were stiffer than hardwood composites probably due to the higher lignin content (ex: spruce (softwood)—28% and aspen (hardwood)—18%).	[19,83]
Hardwood	- Hardwood fibers have an average length of 1.2 mm and a width of 3 μm. - Unlike softwood, hardwood has separate cells that perform the functions of conduction and support. Hardwood is distinguished from softwood by the presence of complex water-conducting cells known as vessel elements. Since these vessel elements are narrow tubes with open ends known as pores, hardwood is referred to as “porous wood”. - As a result of the higher cellulose content, hardwood composites had better tensile strength, impact strength, and elongation.	[19,83]

**Table 4:** Studies on hybrid natural fiber/natural fiber-reinforced polymer composites.

Fiber 1	Fiber 2	Parameter	Matrix Type	Processing Technique	Mechanical Performance	Ref.
Oil palm empty fruit bunch (EFB) fiber mat	Woven jute (Jw)	Layering pattern of hybrid composite: EFB/Jw/EFB and Jw/EFB/Jw	Epoxy	Hand lay-up	- It was found that the tensile and flexural properties of the hybrid composite was higher than that of the non-hybrid composite.	[147]
Banana fibers (B)	Woven coconut sheath (C)	Random composite orientation: CBC, CCB, BCB, BBC, pure banana (BBB), and pure coconut sheath (CCC)	Unsaturated polyester	Compression molding	- There was not much difference in the mechanical properties between the pure coconut sheath and banana hybrid composites. - However, the flexural strength was higher for the coconut sheath hybrid composite. - The mechanical properties were also found to vary with the layering pattern.	[148]
PALF	Kenaf fiber (KF)	Fiber loading: (PF:PALF:KF) 50:50:0, 50:35:15, 50:25:25, 50:15:35, 50:0:50	Phenol formaldehyde	Hand lay-up	- The hybrid composite improved in term of tensile strength (46.96 MPa) and modulus (6.84 GPa), flexural strength (84.21 MPa) and modulus (5.81 GPa), and impact strength (5.39 kJ/m^2^) as compared with the PALF/PF and KF/PF composites. - The hybrid composite also showed the highest storage modulus and loss modulus.	[140,149]
Unidirectional long flax fiber (F)	Woven sugar palm fiber (S)	Fiber stacking sequences: All F, All S, F/F/S/S/F/F and S/F/F/F/F/S	Epoxy	Hot press molding	- Tensile strength and flexural strength of the hybrid composites increased by nearly 3-folds and 2-folds, respectively. - The results from dynamic mechanical analysis and short beam test revealed that the hybridization of F fiber into the S fiber-reinforced composites caused superior mechanical strength, dynamic mechanical properties, and interlaminar shear strength.	[150]
Jute fiber (J) Sisal fiber (S)	Curaua fibers (C)	Fiber hybridization and treatment: Untreated J, treated J, mixed J Untreated J + C, Treated J + C, Mixed J + C Untreated S + C, Treated S+C, Mixed S + C	Epoxy	Hand lay-up	- The tensile, flexural, and impact properties of hybrid composites are significantly improved by the addition of the untreated fibers to J fiber-based composites. - The alkaline treatment had a positive impact on the mechanical properties of the composite, while for the J + C hybrid composites, the alkaline treatment had a negative effect on the tensile and impact properties. The mixed (alkalization + silanization) treatment had a positive effect on the J + C flexural properties, while it decreased the flexural properties for the J + S composite.	[151]
Aloe vera mat (AVM) and flax mat (FM)	Sisal fiber (SF)	Fiber arrangement: AVM-FM-SF-FM-AVM (S1) and FM-AVM-SF-AVM-FM (S2)	Epoxy	Hand lay-up	- The S2 hybrid composites exhibit improved tensile strength, flexural strength, impact strength, and hardness properties. - It is also proven that the hybrid composite formed with FM positioned at the peripheral layers (S2) possesses higher strength in terms of tensile, flexural, impact, and hardness than the hybrid composite formed with AVM at the peripheral layers (S1). - This work also highlights that the flexural and impact property of fiber mats-reinforced in epoxy are moderately superior than that of the neat epoxy material.	[152]
Roselle fiber (RF)	Sugar palm fibers (SPF)	Fiber ratios: 100RF, 70RF:30SPF, 50RF:50SPF, 30RF:70SPF and 100SPF.	PU	Melt mixing and hot compression	- The RF/SPF hybrid composites increased its impact strength corresponding to the increases in the SPF content of the composites. - However, the tensile and flexural properties decreased due to poor interfacial bonding between the fiber and matrix.	[144]
Coir fiber (CF)	PALF	Fiber loading of PLA:CF: PALF (wt %): 100:0:0, 70:30:0, 70:0:30, 70:15:15, 70:9:21, 70:21:9	PLA	Melt mixing method	- The hybrid composites had higher tensile and flexural modulus compared to those of neat PLA. - The strength values were improved upon the addition of PALF, while impact tests showed enhanced strength results upon the addition of CF. - The dynamic mechanical analysis results confirmed that the storage and loss moduli of the hybrid composites increased with respect to those of the neat PLA, whereas the tan δ decreased. - The coefficient of thermal expansion was decreased with the addition of fiber.	[153]
Sisal fiber	Hemp fiber	-	PLA	Melt processing and injection molding	- The achieved mean tensile strength, Young’s modulus, and specific tensile strength of hybrid composites were improved compared to neat PLA. - The flexural modulus and specific flexural strength of hybrid composites also showed better performance than those of neat PLA. - Incorporation of sisal and hemp fiber with PLA remarkably increased the impact strength of composites.	[146]
Wood fiber	Rice husk	Wood content: 10%, 20%, and 30% Rice husk content: 10%, 20%, and 30% Hybrid content: 5%, 10%, and 15%	PP	Injection molding	- The tensile modulus of hybrid composites increased as the filler loading increased. - The flexural strength decreased with the filler loading.	[154]
Woven jute	Woven flax	Fiber ratio: Neat PLA, Jute/PLA, Flax/PLA, and Hybrid Jute Flax/PLA	PLA	Compression molding	- Hybrid composites achieved higher flexural strength and modulus, whereas flax/PLA have higher tensile strength. - For impact strength, the hybrid composite has achieved a higher value. - The hybrid composites have lower dynamic mechanical properties than the other types of composites.	[155]
Kenaf fiber (KF)	Aloe vera fiber (AF)	Composite compositions: PLA, PLA/treated KF, PLA/treated AF, PLA/treated KF/treated AF, PLA/treated KF/treated AF/1MMT and PLA/treated KF/treated AF/3MMT MMT = montmorillonite	PLA	Compression molding	- The mechanical properties were found to be increased upon 15 wt % KF, 15 wt % AF hybridization, and 1 wt % MMT clay incorporated. - The 1 wt % MMT included hybrid composite exhibited increased tensile strength, flexural strength, impact strength, and abrasion resistance compared to virgin PLA. - The tensile and flexural moduli of these composites are improved compared with neat PLA.	[156]

**Table 5:** Summary of works on hybrid natural fiber/synthetic fiber-reinforced polymer composites.

Natural Fiber	Synthetic Fiber	Matrix Type	Processing Technique	Ref.
EFB	Glass	Unsaturated polyester	RTM	[160]
Basalt and flax	Carbon	Epoxy	Hand lay-up and vacuum bagging	[177]
Short basalt	Short fiber PP	Epoxy	Injection molding	[178]
Flax	Carbon	Epoxy	Vacuum-assisted resin transfer molding (VARTM)	[164]
Sisal	Glass	PP	Single extrusion machine and press consolidation	[165]
Vetiveria zizanioides/Jute	Glass	Vinyl ester	Hand lay-up	[168]
EFB	Glass	PP	Extrusion and injection molding	[170]
Areca sheath and jute	Woven-glass	Epoxy	Hand lay-up	[172]
Sugar palm yarn	Woven-glass	Unsaturated polyester	Hand lay-up	[136]
Longitudinal basalt	Woven-glass	Unsaturated polyester-resin	Hand lay-up	[173]
Bamboo powder	Glass	Epoxy	Hand lay-up	[179]
Bamboo	Glass	Epoxy	Curing	[175]
Bamboo	Glass	Unsaturated polyester	Hand lay-up	[176]

**Table 6:** Studies on hybrid natural fiber/metal-reinforced polymer composites.

Natural Fiber	Metal Laminate Type	Matrix Type	Processing Technique	Ref.
Jute fiber	Aluminum and magnesium	Epoxy	Hand lay-up and compression molding	[181]
Plain sisal fabric	Aluminum	Epoxy	Cold pressing	[182]
Unidirectional tape flax fibers and sugar palm fibers	Aluminum alloy	Epoxy	Hand lay-up and hot press	[183]
Kenaf fiber, flax fiber, and carbon fiber	Aluminum alloy	Epoxy	Hand lay-up	[184]
Woven mat jute fiber	Aluminum and copper	Epoxy	Compression molding	[185]
Plain woven kenaf and woven E-glass	Annealed aluminum	PP	Hot pressing	[186]
Plain and twill woven kenaf and PALF	Aluminum	PP	Hot molding compression	[187]

**Table 7:** Studies on hybrid natural fiber/carbonaceous material-reinforced polymer composites.

Carbonaceous Material	Natural Fiber	Matrix Type	Key Findings	Ref.
GO and graphene flakes	Untreated jute fiber and alkaline-treated jute fiber	Epoxy	The Young’s modulus and tensile strength of graphene-based jute fiber composites jute−epoxy composites is increased by ≈324% and ≈110%, respectively, more than untreated jute fiber composites.	[198]
GO	Curaua fiber (CF)	Unsaturated polyester	The tensile and flexural strength of CF/GO-reinforced unsaturated polyester-based composites increased by 156% and 186%, respectively, in comparison to the neat unsaturated polyester.	[199]
GO	Curaua fiber (CF)	Epoxy	The CF/GO epoxy-based composites increased in yield strength by 64%, tensile strength by 40%, Young’s modulus by 60%, and toughness by 28% compared to the CF-reinforced epoxy composite.	[200]
Exfoliated graphite nanoplatelets	Kenaf fiber	PLA	The addition of 5 wt % xGnP increased the flexural modulus by 25 to 30% but did not increase the strength. The addition of xGnP to the heat distortion temperature had a beneficial impact but only at higher fiber loadings.	[201]
Graphene	Bagasse fiber (BS)	PP	Tensile, flexural, and notched impact strength values were greatest in composites containing 0.1 wt % graphene and 30 wt % BF.	[202]
GO	Sisal fiber (SF)	PP	The combined treatment of GO and maleic anhydride-grafted polypropylene (MAPP) improved the mechanical properties, melting temperature, and water resistance of the GO-SF/MAPP-PP composite significantly.	[203]
CNTs	Bamboo fiber	Epoxy	The composite’s mechanical (tensile, flexural, and impact) and water resistance properties increased after CNTs were added. There was a significant increase in impact strength by 84.5%.	[204]
CNTs, acid-treated (ACNT) and acid silane treated (SCNT)	Kenaf fiber	Epoxy	The tensile, flexural, and impact properties of the kenaf/epoxy composite were strengthened by 43.30%, 21.10%, and 130%, respectively, when 1 wt % acid-silane treated CNT was included.	[205]
MWCNTs)	Cotton cellulose nanofiber (CNF-C)	PU	The PU matrix completely cross-linked with CNF-C and CNTs demonstrated good mechanical properties and sensing efficiency. The hybrid composite can accurately sense massive strains more than 103 times, and water-induced form recovery can help to sustain sensing precision after material fatigue.	[206]
MWCNTs	Oil palm shell (OPS)	Unsaturated polyester	It was discovered that a small amount of pristine MWCNTs dispersed inside the natural filler unsaturated polyester composite may improve the mechanical properties of the hybrid composite.	[207]

**Table 8:** Automotive models and their components implementing natural fiber composites.

Models	Brands	Components
C3 Picasso, C5	Citroen	Boot linings, mud guards, interior door paneling, parcel shelves, and door panels
Passat Variant, Golf, A4, Bora	Volkswagen	Door panel, boot-liner, seat back, and boot-lid finish panel
Vectra, Astra, Zafira	Opel	Head-liner panel, pillar cover panel, door panels, and instrumental panel
3, 5 and 7 series	BMW	Noise insulation panels, headliner panel, seat back, door panels, molded foot well linings, and boot-lining
Mondeo CD 162, Focus	Ford	Floor trays, door inserts, door panels, B-pillar, and boot-liner
C70, V70	Volvo	Seat padding, natural foams, cargo floor tray, dash, boards and ceilings
Eco Elise	Lotus	Seats, interior carpets, body panels, and spoiler,
ES3	Toyota	Pillar garnish and other interior parts
2000	Rover	Rear storage shelf/panel, and insulations
Fiat SpA	Mitsubishi	Indoor cladding, seat back, cargo area floor, door panels, lining, instrumental panel, floor mats, and floor panels
406	Peugeot	Seat backs, parcel shelf, front and rear door panels
A, C, E, and S class	Daimler Chrysler	Pillar cover panel, door panels, car windshield/car dashboard, and business table

## Data Availability

Not applicable.
